# Lys-urea-Aad, Lys-urea-Cmc and Lys-urea-Cms as potential pharmacophores for the design of PSMA-targeted radioligands to reduce off-target uptake in kidneys and salivary glands

**DOI:** 10.7150/thno.87663

**Published:** 2023-08-15

**Authors:** Hsiou-Ting Kuo, Zhengxing Zhang, Chengcheng Zhang, Helen Merkens, Ruiyan Tan, Antonio A. W. L. Wong, Carlos F. Uribe, François Bénard, Kuo-Shyan Lin

**Affiliations:** 1 Department of Molecular Oncology, BC Cancer, Vancouver, BC V5Z1L3, Canada; 2 Department of Functional Imaging, BC Cancer, Vancouver, BC V5Z4E6, Canada; 3 Department of Radiology, University of British Columbia, Vancouver, BC V5Z1M9, Canada

**Keywords:** Prostate-specific membrane antigen, Targeted radioligand therapy, Salivary gland, Off-target uptake, Tumor-to-kidney absorbed dose ratio

## Abstract

High kidney and salivary gland uptake is a common feature of prostate-specific membrane antigen (PSMA)-targeted radioligands derived from the lysine-urea-glutamic acid (Lys-urea-Glu) pharmacophore. In this study we investigated if radioligands derived from lysine-urea-2-aminoadipic acid (Lys-urea-Aad), lysine-urea-*S*-carboxylmethylcysteine (Lys-urea-Cmc) and lysine-urea-*O*-carboxylmethylserine (Lys-urea-Cms) pharmacophores with/without an albumin binder could retain good PSMA-targeting capability but with minimized kidney and salivary gland uptake.

**Methods:** HTK03177 and HTK03187 were obtained by replacing Aad in the previously reported

Lys-urea-Aad-derived HTK03149 with Cmc and Cms, respectively. HTK03170, HTK04048 and HTK04028 were derived from HTK03149, HTK03177 and HTK03187, respectively, with the conjugation of an albumin-binding moiety, 4-(*p*-methoxyphenyl)butyric acid. *In vitro* competition binding assays were conducted using PSMA-expressing LNCaP prostate cancer cells and [^18^F]DCFPyL as the radioligand. Imaging and biodistribution studies of ^68^Ga-labeled HTK03177 and HTK03187, and ^177^Lu-labeled HTK03170, HTK04048 and HTK04028 were performed in LNCaP tumor-bearing mice. Radioligand therapy study of [^177^Lu]Lu-HTK03170 was carried out in LNCaP tumor-bearing mice and [^177^Lu]Lu-PSMA-617 was used for comparison.

**Results:** The calculated K_i_(PSMA) values of Ga-HTK03177, Ga-HTK03187, Lu-HTK03170, Lu-HTK04048 and Lu-HTK04028 were 5.0±2.4, 10.6±2.0, 1.6±0.4, 1.4±1.0 and 13.9±3.2 nM, respectively. PET Imaging and biodistribution studies at 1 h post-injection showed that both [^68^Ga]Ga-HTK03177 and [^68^Ga]Ga-HTK03187 had high uptake in LNCaP tumor xenografts (24.7±6.85 and 21.1±3.62 %ID/g, respectively) but minimal uptake in normal organs/tissues including kidneys (7.76±1.00 and 2.83±0.45 %ID/g, respectively) and salivary glands (0.22±0.02 and 0.16±0.02 %ID/g, respectively). SPECT imaging and biodistribution studies showed that the LNCaP tumor uptake of ^177^Lu-labeled HTK03170, HTK04048 and HTK04028 peaked at 4-24 h post-injection at ~43-65 %ID/g and was relatively sustained over time. Their peaked average uptake in kidneys (≤ 17.4 %ID/g) and salivary glands (≤ 2.92 %ID/g) was lower and continuously reduced over time. Radioligand therapy study showed that compared with [^177^Lu]Lu-PSMA-617 (37 MBq), a quarter dose of [^177^Lu]Lu-HTK03170 (9.3 MBq) led to a better median survival (63 vs 90 days).

**Conclusions:** Our data demonstrate that that Lys-urea-Aad, Lys-urea-Cmc and Lys-urea-Cms are promising pharmacophores for the design of PSMA-targeted radioligands especially for radiotherapeutic applications to minimize toxicity to kidneys and salivary glands.

## Introduction

**Figure 1:**
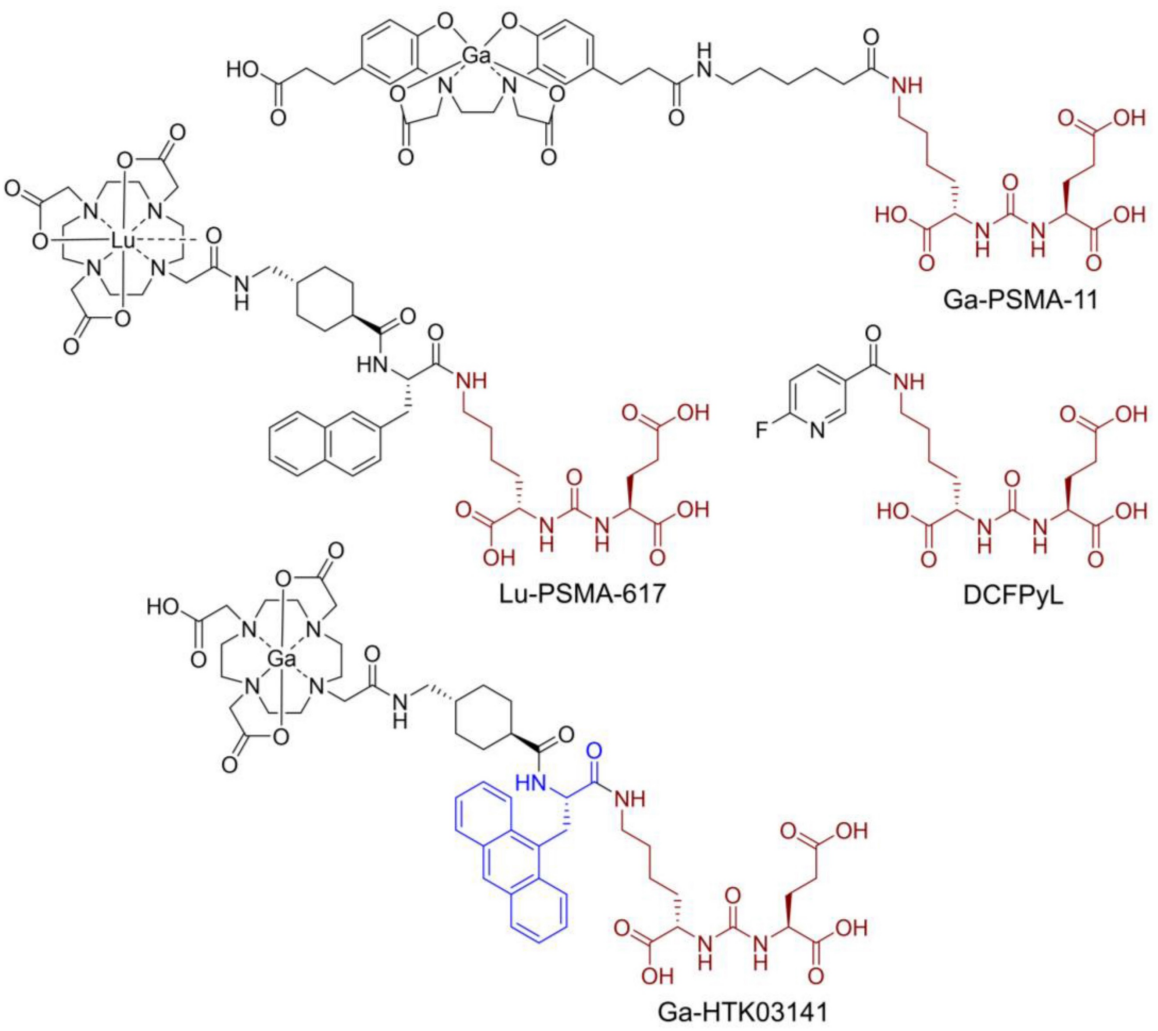
Chemical structures of reported PSMA-targeted ligands. The PSMA-targeting Lys-urea-Glu pharmacophore is in brown, and the 9-anthrylalanine in Ga-HTK03141 is in blue.

Prostate-specific membrane antigen (PSMA), also known as glutamate carboxypeptidase II, is a transmembrane enzyme and is highly expressed on prostate cancer cells 1. Its expression is positively correlated with disease progression and worst prognosis 2. PSMA is not expressed in most normal organs/tissues and is, therefore, a promising diagnostic maker and therapeutic target for the management of prostate cancer 3-4. Various PSMA-targeted radioligands have been successfully validated in the clinic for imaging and radioligand therapy of prostate cancer. The reported PSMA-targeted radioligands are mainly based on a lysine-urea-glutamic acid (Lys-urea-Glu) pharmacophore, including the US FDA-approved [^68^Ga]Ga-PSMA-11 5, [^18^F]DCFPyL 6 and [^177^Lu]Lu-PSMA-617 7 (Figure 1). The Lys-urea-Glu-derived radioligands often lead to high and sustained off-target uptake in normal organs such as kidneys and salivary glands. The high and sustained off-target uptake of PSMA-targeted radioligands in normal organs reduce their sensitivity for detecting lesions in and adjacent to these organs 8, and could potentially cause toxicity especially when radiolabeled with an α-emitter such as ^225^Ac for radioligand therapy 9.

To improve tumor uptake and potentially reduce off-target uptake, we have evaluated several ^68^Ga-labeled PSMA-617 derivatives by replacing the 2-naphthylalanine (2-Nal) moiety in PSMA-617 with various aromatic amino acids 10-11. [^68^Ga]Ga-HTK03141 (Figure 1) with a 9-anthrylalanine moiety showed enhanced PSMA binding affinity and higher uptake in PSMA-expressing LNCaP tumor xenografts when compared with [^68^Ga]Ga-PSMA-617 11. However, very high uptake of [^68^Ga]Ga-HTK03141 in mouse kidneys (170±26.4 %ID/g, 1 h post-injection) and salivary glands (4.99±0.88 %ID/g, 1 h post-injection, unreported data) was also observed 11.

**Figure 2:**
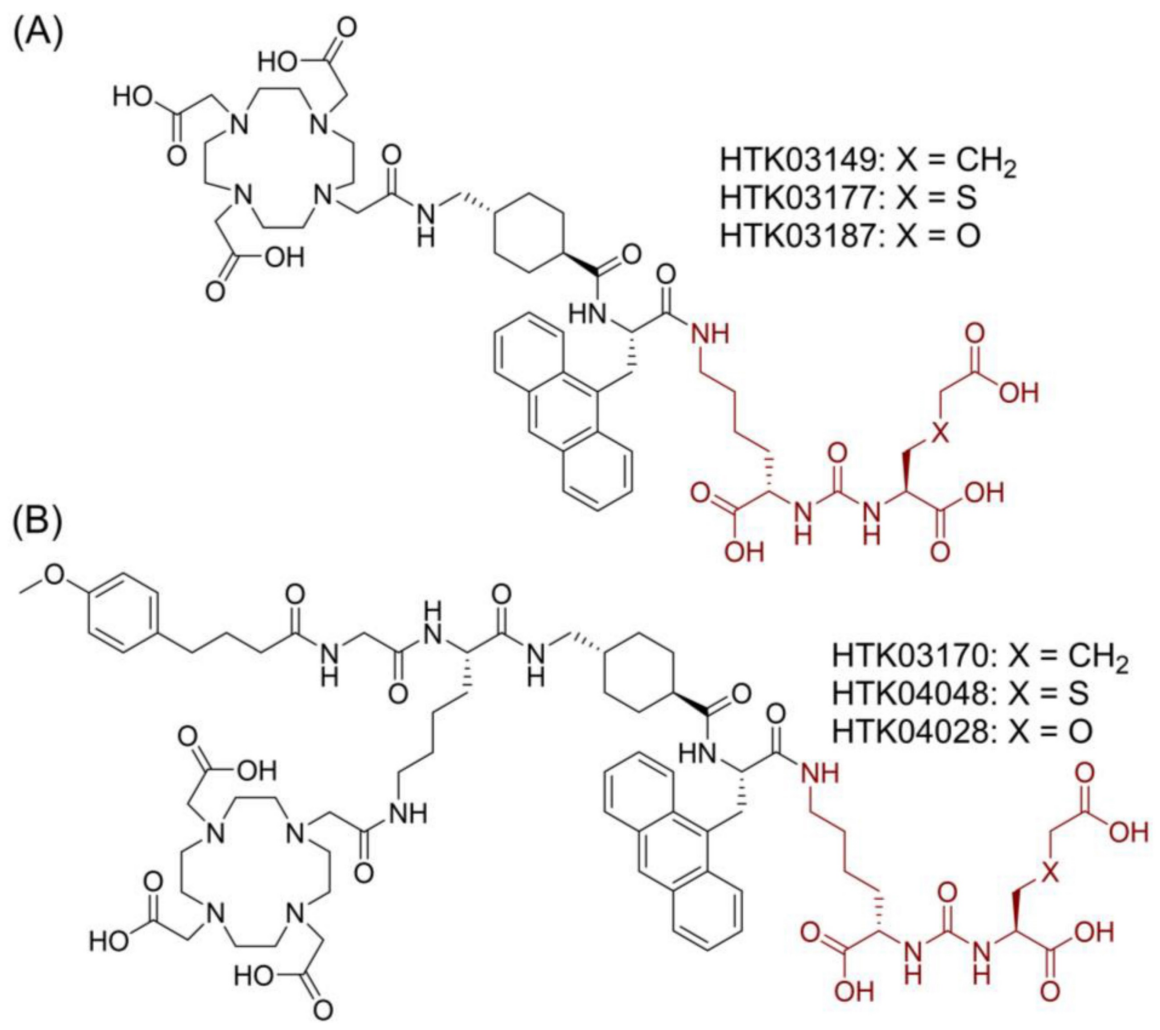
Chemical structures of (A) HTK03149, HTK03177 and HTK03187; and (B) HTK03170, HTK04048 and HTK04028. The novel PSMA-targeting pharmacophores (X = CH_2_: Lys-urea-Aad; X = S: Lys-urea-Cmc; X = O: Lys-urea-Cms) are in brown.

Suspecting that the high kidney and salivary gland uptake of reported PSMA-targeted radioligands might be due to the Glu moiety in the Lys-urea-Glu pharmacophore, we subsequently investigated if replacing Glu with a close analog could lead to new derivatives with reduced off-target uptake in normal organs/tissues 12. [^68^Ga]Ga-HTK03149 (X = CH_2_, Figure 2A) is a derivative of [^68^Ga]Ga-HTK03141 by replacing Glu in the PSMA-targeting Lys-urea-Glu pharmacophore with 2-aminoadipic acid (Aad). Compared with [^68^Ga]Ga-HTK03141, [^68^Ga]Ga-HTK03149 showed comparable uptake in PSMA-expressing tumor xenografts (23.1±6.11 vs 19.1±6.37 %ID/g, 1 h post-injection), but with greatly reduced uptake in normal organs/tissues including kidneys (170±26.4 vs 4.15±1.46 %ID/g, 1 h post-injection) and salivary glands (4.99±0.88 vs 0.22±0.06 %ID/g, 1 h post-injection) 12.

In this study, we investigated the effect of replacing Aad in HTK03149 with a close analog, *S*-carboxylmethylcysteine (Cmc in HTK03177, X = S, Figure 2A) and *O*-carboxymethylserine (Cms in HTK03187, X = O, Figure 2A) on the PSMA targeting capability of their Ga-complexed analogs by *in vitro* binding assay, PET imaging and *ex vivo* biodistribution studies. We also compared the PSMA-targeting capability of the Lu-complexed and albumin-binder-conjugated analogs (Figure 2B) of HTK03149 (HTK03170), HTK03177 (HTK04048) and HTK03187 (HTK04028) by *in vitro* binding assay, SPECT imaging and *ex vivo* biodistribution studies. Finally, [^177^Lu]Lu-HTK03170 was selected as our lead candidate, and its treatment efficacy was demonstrated by radioligand therapy study in mice bearing PSMA-expressing LNCaP tumor xenografts, and compared with [^177^Lu]Lu-PSMA-617.

## Materials and Methods

### General methods

All chemicals and solvents were obtained from commercial sources, and used without further purification. PSMA-targeted peptides were synthesized using solid phase approach on an AAPPTec (Louisville, KY, USA) Endeavor 90 peptide synthesizer. Purification and quality control of nonradioactive and radiolabeled peptides were performed using one of the Agilent (Santa Clara, CA, USA) HPLC systems: (A) a model 1260 Infinity II preparative binary pump, a model 1260 Infinity variable wavelength detector (set at 220 nm), and a 1290 Infinity II preparative open-bed fraction collector; (B) a model 1260 Infinity quaternary pump and a model 1200 variable wavelength detector (set at 220 nm); and (C) a model 1200 quaternary pump, a model 1200 UV absorbance detector (set at 220 nm), and a Bioscan (Washington, DC) NaI scintillation detector. The HPLC columns used were (A) a preparative column (Gemini, NX-C18, 5 µ, 50 × 30 mm); (B) a semi-preparative column (Luna C18, 5 µ, 250 × 10 mm); and (C) an analytical column (Luna C18, 5 µ, 250 × 4.6 mm) purchased from Phenomenex (Torrance, CA, USA). The collected HPLC eluates containing the desired peptide were lyophilized using a Labconco (Kansas City, MO, USA) FreeZone 4.5 Plus freeze-drier. Mass analyses were performed using an AB SCIEX (Framingham, MA) 4000 QTRAP mass spectrometer system with an ESI ion source. C18 Sep-Pak cartridges (1 cm^3^, 50 mg) were obtained from Waters (Milford, MA, USA). ^68^Ga was eluted from an iThemba Labs (Somerset West, South Africa) generator, and purified according to the previously published procedures using a DGA resin column from Eichrom Technologies LLC (Lisle, IL, USA) 13. [^177^Lu]LuCl_3_ solution was ordered from ITG Isotope Technologies Garching GmBH (Garching, Germany). Radioactivity of ^68^Ga- and ^177^Lu-labeled peptides was measured using a Capintec (Ramsey, NJ, USA) CRC^®^-25R/W dose calibrator, and the radioactivity of mouse tissues collected from biodistribution studies were counted using a Perkin Elmer (Waltham, MA, USA) Wizard2 2480 automatic gamma counter.

### Synthesis of Fmoc-L-Cmc(O*t*Bu)-O*t*Bu (1)

*S*-Carboxymethyl-L-cysteine (Cmc, 1.79 g, 10 mmol) and NaHCO_3_ (2.10 g, 25 mmol) in water (40 mL) was added FmocOSu (3.37 g, 10 mmol) in 1,4-dioxane (40 mL). The resulting solution was stirred at room temperature for 20 h. After washing with diethyl ether (100 mL × 2), the aqueous phase was acidified with concentrated HCl to pH 2. The acidic solution was extracted with ethyl acetate (100 mL × 2), and the organic phases were combined, dried over anhydrous MgSO_4_, and evaporated to give 4.93 g crude Fmoc-protected Cmc as a sticky solid.

The crude Fmoc-protected Cmc (4.93 g) in CH_2_Cl_2_ (30 mL) was added *tert*-​butyl 2,​2,​2-​trichloroacetimidate (8.74 g, 40 mmol). The resulting mixture was stirred at room temperature for 17 h, and then purified by flash column chromatography eluted with 1:4 diethyl ether/hexanes to 1:2 diethyl ether/hexanes to obtain **1** (4.61 g, 90%) as a colorless thick oil. ^1^H NMR (300 MHz, CDCl_3_) δ 7.77 (d, *J* = 7.5 Hz, 4H), 7.62 (d, *J* = 7.3 Hz, 4H), 7.39 (td, *J* = 7.5, 1.3 Hz, 4H), 7.31 (td, *J* = 7.5, 1.3 Hz, 4H), 5.83 (d, *J* = 7.9 Hz, 1H), 4.53 (q, *J* = 5.6 Hz, 2H), 4.39 (d, *J* = 7.3 Hz, 4H), 4.24 (t, *J* = 7.1 Hz, 2H), 3.28-3.00 (m, 6H), 1.49 (s, 9H), 1.48 (s, 9H). MS (ESI) calculated [M + Na]^+^ for C_28_H_35_NO_6_SNa 536.2, found 536.0.

### Synthesis of L-Cmc(O*t*Bu)-O*t*Bu.HCl (2)

Compound **1** (4.61 g, 9.0 mmol) in DMF (40 mL) was added NaN_3_ (715 mg, 11 mmol). The resulting solution was heated at 50 °C for 20 h. After cooling down, the reaction mixture was mixed with 2 N HCl (8 mL) and water (80 mL), and then washed with ethyl acetate (100 mL × 2). The aqueous phase was neutralized with sodium bicarbonate powder and extracted with ethyl acetate (100 mL × 2). The ethyl acetate fractions were combined, dried over anhydrous MgSO_4_, and evaporated under reduced pressure. The crude product was dissolved in diethyl ether (150 mL), and the resulting solution was added with 4 N HCl in 1,4-dioxane (5 mL). The resulting solution was stirred for 5 min, and the precipitate was collected to give **2** (2.09 g, 71%) as a white powder. ^1^H NMR (300 MHz, CDCl_3_) δ 4.34 (t, *J* = 5.3 Hz, 1H), 3.54 - 3.33 (m, 5H), 1.51 (s, 9H), 1.47 (s, 9H). MS (ESI) calculated [M + H]^+^ for C_13_H_26_NO_4_S 292.2, found 292.1.

### Synthesis of Fmoc-L-Cms(O*t*Bu)-O*t*Bu (3)

Side chain *t*-butyl-protected *O*-carboxymethyl-L-serine (Cms, 1.02 g, 4.6 mmol) and NaHCO_3_ (840 mg, 10 mmol) in water (30 mL) was added FmocOSu (1.56 g, 4.6 mmol) in 1,4-dioxane (30 mL). The resulting solution was stirred at room temperature for 25 h. After diluting with brine (50 mL), the mixture was acidified to pH 3 with 1 N HCl. The acidic solution was extracted with ethyl acetate (50 mL × 2), and the organic phases were combined, dried over anhydrous MgSO_4_, and evaporated to give 2.47 g crude Fmoc-L-Cms(O*t*Bu)-OH as a sticky solid.

The crude Fmoc-L-Cms(O*t*Bu)-OH (2.47g) in CH_2_Cl_2_ (30 mL) was added *tert*-​butyl 2,​2,​2-​trichloroacetimidate (2.18 g, 10 mmol). The resulting mixture was stirred at room temperature for 22 h, and then purified by flash column chromatography eluted with 1:2 diethyl ether/hexanes to obtain **3** (2.10 g, 92%) as a colorless thick oil. ^1^H NMR (300 MHz, CDCl_3_) δ 7.76 (d, *J* = 7.5 Hz, 2H), 7.65 (dd, *J* = 7.5, 4.1 Hz, 2H), 7.40 (td, *J* = 7.5, 1.3 Hz, 2H), 7.31 (td, *J* = 7.4, 1.3 Hz, 2H), 6.15 - 6.05 (m, 1H), 4.47 - 4.29 (m, 3H), 4.25 (t, *J* = 7.3 Hz, 1H), 4.08 - 3.94 (m, 3H), 3.78 (dd, *J* = 9.4, 3.0 Hz, 1H), 1.50 (s, 9H), 1.49 (s, 9H). MS (ESI) calculated [M + Na]^+^ for C_28_H_35_NO_7_Na 520.2, found 520.2.

### Synthesis of L-Cms(O*t*Bu)-O*t*Bu.HCl (4)

Compound **3** (2.05 g, 4.1 mmol) and Pd/C (10%, 300 mg) in methanol (70 mL) was hydrogenated using a balloon for 22 h. After filtration through Celite, the solvent was removed *in vacuo*. The residue was dissolved in diethyl ether (200 mL) and 3 mL of 4 N HCl in 1,4-dioxane was added. The resulting solution was put into freezer overnight, and the resulting precipitate was collected to give **4** (1.18 g, 92%) as a white solid. ^1^H NMR (300 MHz, DMSO) δ 8.52 (s, 3H), 4.16 (t, *J* = 3.5 Hz, 1H), 4.06 (d, *J* = 7.7 Hz, 2H), 3.97 (dd, *J* = 10.8, 4.2 Hz, 1H), 3.84 (dd, *J* = 10.6, 3.1 Hz, 1H), 1.46 (s, 9H), 1.43 (s, 9H). MS (ESI) calculated [M + H]^+^ for C_13_H_26_NO_5_ 276.2, found 276.1.

### Synthesis of DOTA-conjugated PSMA-targeted ligands

HTK03177, HTK03187, HTK03170, HTK04048 and HTK04028 were synthesized by solid-phase peptide chemistry. Fmoc-Lys(ivDde)-Wang resin (0.1 mmol, 0.58 mmol/g loading) was suspended in DMF for 30 min. Fmoc was then removed by treating the resin with 20% piperidine in DMF (3 × 8 min). To generate the isocyanate derivative, a solution of Aad di-*tert*-butyl ester hydrochloride 12 (155 mg, 0.5 mmol, 5 eq relative to resin), Cmc di-*tert*-butyl ester hydrochloride (**2**, 164 mg, 0.5 mmol, 5 eq relative to resin), or Cms di-*tert*-butyl ester hydrochloride (**4**, 156 mg, 0.5 mmol, 5 eq relative to resin), and DIEA (287 µL, 1.65 mmol) in CH_2_Cl_2_ (5 mL) was cooled to -78 °C in a dry ice/acetone bath. Triphosgene (49 mg, 0.17 mmol) was dissolved in CH_2_Cl_2_ (5 mL), and the resulting solution was added dropwise to the reaction at -78 °C. The reaction was then allowed to warm to room temperature and stirred for 30 minutes to give a solution of the isocyanate derivative. After which another 87 µL DIEA (0.5 mmole) was added, and then added to the lysine-immobilized resin and reacted for 16 h. After washing the resin with DMF, the ivDde-protecting group was removed with 2% hydrazine in DMF (5 × 5 min). Fmoc-Ala(9-Anth)-OH (4 eq.) was then coupled to the side chain of Lys followed by Fmoc-tranexamic acid and DOTA-tris(*t*-butyl)ester for HTK03177 and HTK03178. For albumin-binder-containing peptidomimetics HTK03170, HTK04048 and HTK04028, after coupling with Fmoc-tranexamic acid, elongation was continued with the addition of Fmoc-Lys(ivDde)-OH, Fmoc-Gly-OH, and 4-(*p*-methoxyphenyl)butyric acid using Fmoc-based chemistry. All coupling were carried out in DMF using Fmoc-protected amino acid (4 eq.), HATU (4 eq.), and DIEA (7 eq.). After removal of the ivDde-protecting group with 2% hydrazine in DMF (5 × 5 min), DOTA-tris(*t*-butyl)ester was then coupled to the side chain of Lys to give the radiolabeling precursors.

The peptides were deprotected and simultaneously cleaved from the resin by treating with 95/5 trifluoroacetic acid (TFA)/triisopropylsilane for 2 h at room temperature. After filtration, the peptides were precipitated by the addition of cold diethyl ether to the TFA solution. The crude peptides were purified by HPLC. The eluates containing the desired peptides were collected, pooled, and lyophilized. The HPLC conditions, retention times, isolated yields and MS confirmations of these DOTA-conjugated ligands are provided in Table S1.

### Synthesis of nonradioactive Ga-complexed standards

To prepare nonradioactive Ga-complexed standards, a solution of the DOTA-conjugated precursor was incubated with GaCl_3_ (5 eq.) in NaOAc buffer (0.1 M, 500 µL, pH 4.2) at 80 °C for 15 min. The reaction mixture was then purified by HPLC, and the HPLC eluates containing the desired peptide were collected, pooled, and lyophilized. The HPLC conditions, retention times, isolated yields and MS confirmations of these nonradioactive Ga-complexed standards are provided in Table S2.

### Synthesis of nonradioactive Lu-complexed standards

To prepare the nonradioactive Lu-complexed standards, a solution of the DOTA-conjugated precursor was incubated with LuCl_3_ (5 eq.) in NaOAc buffer (0.1 M, 500 µL, pH 4.2) at 95 °C for 15 min. The reaction mixture was then purified by HPLC, and the HPLC eluates containing the desired peptide were collected, pooled, and lyophilized. The HPLC conditions, retention times, isolated yields and MS confirmations of nonradioactive Lu-complexed standards are provided in Table S2.

### Synthesis of ^68^Ga-labeled PSMA-targeted ligands

The radiolabeling experiments were performed following previously published procedures 13. Purified [^68^Ga]GaCl_3_ in 0.5 mL water was added into a 4‐mL glass vial preloaded with 0.7 mL of HEPES buffer (2 M, pH 5.0) and 50 μg precursor. The radiolabeling reaction was carried out under microwave heating for 1 min. The reaction mixture was purified by HPLC using the semi‐preparative column. The eluate fraction containing the radiolabeled product was collected, diluted with water (50 mL), and passed through a C18 Sep-Pak cartridge that was pre-washed with ethanol (10 mL) and water (10 mL). After washing the C18 Sep-Pak cartridge with water (10 mL), the ^68^Ga-labeled product was eluted off the cartridge with ethanol (0.4 mL), and diluted with phosphate-buffered saline (PBS) for imaging and biodistribution studies. Quality control was performed using the analytical column. The HPLC conditions and retention times are provided in Table S3.

### Synthesis of ^177^Lu-labeled PSMA-targeted ligands

[^177^Lu]LuCl_3_ (~500 MBq in ~15 μL) was added to a solution of the DOTA-conjugated precursor (25 μg) in NaOAc buffer (0.5 mL, 0.1 M, pH 4.5). The mixture was incubated at 90 °C for 15 min, and then purified by HPLC using the semi‐preparative column. The eluate fraction containing the radiolabeled product was collected, diluted with water (50 mL), and passed through a C18 Sep-Pak cartridge that was pre-washed with ethanol (10 mL) and water (10 mL). After washing the C18 Sep-Pak cartridge with water (10 mL), the ^177^Lu-labeled product was eluted off the cartridge with ethanol (0.4 mL), and diluted with PBS for imaging and biodistribution studies. Quality control was performed using the analytical column. The HPLC conditions and retention times are provided in Table S3.

### Cell culture

The LNCaP cells obtained from ATCC (via Cedarlane, Burlington, Canada) were cultured in RPMI 1640 medium supplemented with 10% FBS, penicillin (100 U/mL) and streptomycin (100 μg/mL) at 37 °C in a Panasonic Healthcare (Tokyo, Japan) MCO-19AIC humidified incubator containing 5% CO_2_. The cells were confirmed pathogen free by the IMPACT Rodent Pathogen Test (IDEXX BioAnalytics). Cells grown to 80-90% confluence were then washed with sterile PBS and collected after trypsinization. The cell concentration was counted in triplicate using a hemocytometer and a manual laboratory counter.

### *In vitro* competition binding assays

*In vitro* competition binding assays were conducted as previously reported using LNCaP prostate cancer cells and [^18^F]DCFPyL as the radioligand 10-12,14. Briefly, LNCaP cells (400,000/well) were plated onto a 24-well poly-D-lysine coated plate for 48 h. Growth media was removed and replaced with HEPES buffered saline (50 mM HEPES, pH 7.5, 0.9% sodium chloride) and the cells were incubated for 1 h at 37 °C. [^18^F]DCFPyL (0.1 nM) was added to each well (in triplicate) containing various concentrations (0.5 mM - 0.05 nM) of tested ligands. Non-specific binding was determined in the presence of 10 µM non-radiolabeled DCFPyL. The assay mixtures were further incubated for 1 h at 37 °C with gentle agitation. Then, the buffer and hot ligand were removed, and cells were washed twice with cold HEPES buffered saline. To harvest the cells, 400 µL of 0.25% trypsin solution was added to each well. Radioactivity was measured on an automatic gamma counter. Nonlinear regression analyses and K_i_ calculations were performed using the GraphPad Prism 7 software.

### Biodistribution, imaging and radioligand therapy studies

Imaging, biodistribution and radioligand therapy experiments were performed following previously published procedures using male NOD-*scid* IL2Rg^null^ (NSG) or NOD.Cg-Rag1^tm1Mom^Il2rg^tm1Wjl^/SzJ (NRG) mice 10-12,14-15. The experiments were conducted according to the guidelines established by the Canadian Council on Animal Care and approved by Animal Ethics Committee of the University of British Columbia. The mice were briefly sedated by inhalation of 2% isoflurane in oxygen, and implanted subcutaneously with 1×10^7^ LNCaP cells behind the left shoulder. The mice were imaged or used in biodistribution studies when the tumor grew to 5-8 mm in diameter over a period of 4-5 weeks.

PET/CT imaging experiments were conducted using a Siemens (Knoxville, TN, USA) Inveon micro PET/CT scanner. Each tumor-bearing mouse was injected with ~6-8 MBq of ^68^Ga-labeled tracer through a lateral caudal tail vein. After 50 min post-injection, a 10-min CT scan was conducted first for localization and attenuation correction after segmentation for reconstructing the PET images, followed by a 10-min static PET imaging acquisition.

SPECT/CT imaging experiments were conducted using the MILabs (Utrecht, The Netherlands) U-SPECT-II/CT scanner. Each tumor-bearing mouse was injected with ∼37 MBq of ^177^Lu-labeled ligand through a lateral caudal tail vein. The mice were imaged at 1, 4, 24, 72, and 120 h after injection. At each time point, a 5-min CT scan was conducted first for anatomical reference followed by 2 × 30-min static emission scans acquired in list mode.

For biodistribution studies, the mice were injected with the radioligand (2-4 MBq) as described above. For blocking, the mice were co-injected with nonradioactive DCFPyL (0.5 mg). At predetermined time points, the mice were sedated by isoflurane inhalation and euthanized by CO_2_ inhalation. Blood was withdrawn by cardiac puncture, and organs/tissues of interest were collected, weighed and counted using an automatic gamma counter (Tables S4-S7).

For radiotherapy study, tumor-bearing mice were injected with saline (the control group), [^177^Lu]Lu-PSMA-617 (37 MBq) or [^177^Lu]-HTK03170 (37, 18.5 and 9.3 MBq) (n = 7-8 per treatment group). Tumor size and body weight were measured twice a week from the date of injection (Day 0) until completion of the study (Day 180). Endpoint criteria were defined as > 20% weight loss, tumor volume > 1000 mm^3^, or active ulceration of the tumor.

### Radiation dosimetry calculation

Internal dosimetry estimates were calculated using the organ level internal dose assessment (OLINDA) software v.2.2 following our previously published procedures 12. These estimates were performed for the mouse using the 25g MOBY phantom 16, and for the tumors using the previously reported unit density sphere model 17. Both the phantom and the sphere model are available in OLINDA and require the input of the total number of decays normalized by injected activity in units of MBq×h/MBq for each of the source organ/tumor.

The biodistribution data (available in Table S5-S7) were used to determine the kinetics input values required by OLINDA. First, each of the values was decayed to its corresponding time point (the values on the table are shown at injection time). Then the different time-points of the uptake data (%ID/g) for each organ were fitted to both mono-exponential (%ID/g = ae^⎯bt^) and bi-exponential (%ID/g = ae^⎯bt^ + ce^⎯dt^) functions using in-house software developed in Python. The best fit was selected based on maximizing the coefficient of determination (R^2^) of the fit and minimizing the residuals. The areas under the curves were analytically calculated based on the parameters obtained from the best fit of each organ and this provided the kinetic input values required by OLINDA.

In the mouse case, the adrenals, blood, fat, and muscle are not modeled in the phantom. These organs were grouped together and included in what OLINDA calls the *remainder of the body*. Lastly, the numbers of decays in the tumors were also calculated based on the biodistribution data of the mice and the values were inputted into the sphere model available in OLINDA.

### Statistical analysis

Statistical analysis was performed by Student's *t*-test using the Microsoft (Redmond, WA, USA) Excel software. The unpaired, one-tailed test was used to compare organ/tissue uptake of [^68^Ga]Ga-HTK03177 and [^68^Ga]Ga-HTK03187 with/without co-injection of DCFPyL (0.5 mg). The difference was considered statistically significant when* p* value was < 0.05.

## Results

### Synthesis of PSMA-targeted ligands

**Scheme 1:**
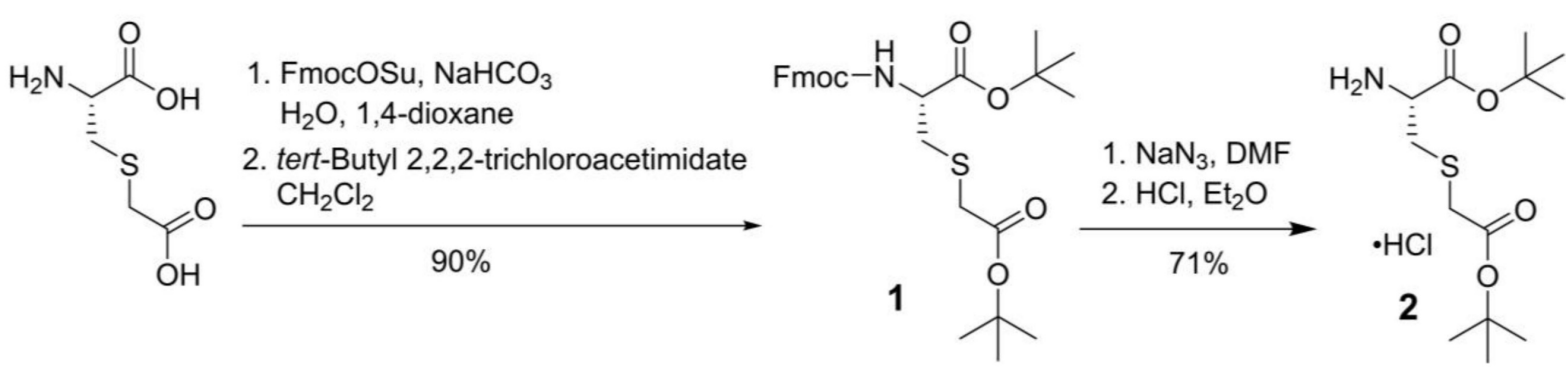
Synthesis of L-Cmc(O*t*Bu)-O*t*Bu.HCl (**2**).

For the synthesis of PSMA-targeted ligands derived from the Lys-urea-Cmc pharmacophore, an intermediate, L-Cmc(O*t*Bu)-O*t*Bu **2**, was synthesized first following the procedures depicted in Scheme 1. Commercially available Cmc was protected with Fmoc at its amino group using FmocOSu, followed by protecting its two carboxylic groups with *t*-butyl using *tert*-​butyl 2,​2,​2-​trichloroacetimidate. Fmoc-L-Cmc(O*t*Bu)-O*t*Bu** 1** was obtained in 90% yield over two steps. Fmoc deprotection by the use of NaN_3_ in DMF, followed by the treatment of the crude product with dry HCl in diethyl ether afforded the desired L-Cmc(O*t*Bu)-O*t*Bu.HCl** 2** in 71% yield over two steps.

**Scheme 2:**
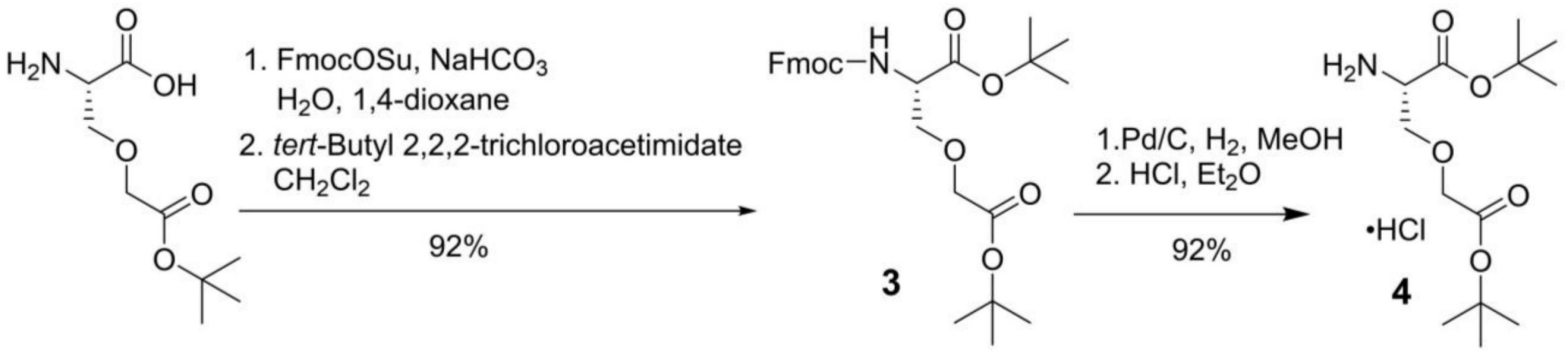
Synthesis of L-Cms(OtBu)-OtBu.HCl (**4**)

Similarly, for the synthesis of Lys-urea-Cms-derived PSMA-targeted ligands, an intermediate, L-Cms(O*t*Bu)-O*t*Bu **4**, was synthesized first following the procedures depicted in Scheme 2. Side chain *t*-butyl-protected Cms was first protected with Fmoc at its amino group using FmocOSu, followed by protecting the remaining free carboxylic group with *t*-butyl using *tert*-​butyl 2,​2,​2-​trichloroacetimidate. Fmoc-L-Cmc(O*t*Bu)-O*t*Bu** 3** was obtained in 92% yield over two steps. Fmoc deprotection by Pd/C-catalyzed hydrogenation 18, followed by the treatment of the crude product with dry HCl in diethyl ether afforded L-Cms(O*t*Bu)-O*t*Bu.HCl** 4** in 92% yield over two steps.

The synthesis of DOTA-conjugated PSMA-targeted ligands was constructed on solid phase using Fmoc chemistry. After cleavage and HPLC purification, HTK03177, HTK03187, HTK03170, HTK04048 and HTK04028 were obtained in 4-53% yield (Table S1). The preparation of nonradioactive Ga- and Lu-complexed standards was conducted in NaOAc buffer (0.1 M, pH 4.2) with excess metal (5 eq.) at elevated reaction temperature. After HPLC purification, the desired Ga- and Lu-complexed standards were obtained in 29-95% yield (Table S2).

^68^Ga labeling was conducted in HEPES buffer (2 M, pH 5.0) under microwave heating for 1 min. After HPLC purification (Table S3), the desired ^68^Ga-labeled HTK03177 and HTK03187 were obtained in 68-75% decay-corrected radiochemical yield with > 50 GBq/µmol molar activity and > 99% radiochemical purity. ^177^Lu labeling was carried out in NaOAc buffer (0.1 M, pH 4.5) at 90 °C for 15 min. After HPLC purification (Table S3), the desired ^177^Lu-labeled HTK03170, HTK04048 and HTK04028 were obtained in 10-49% decay-corrected radiochemical yields with > 55 GBq/µmol molar activity and ≥ 92% radiochemical purity.

### PSMA binding affinities

**Figure 3:**
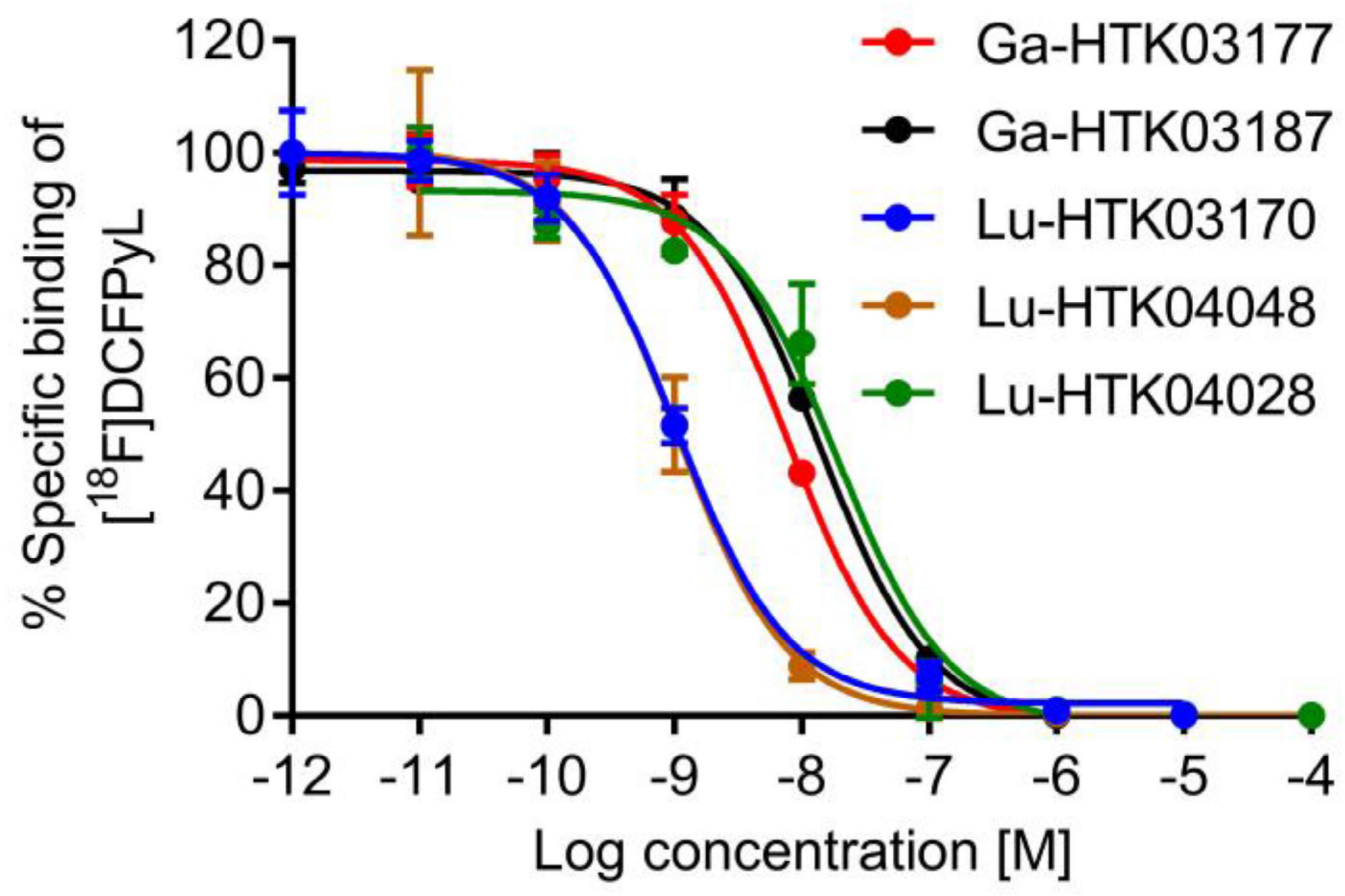
Representative displacement curves of [^18^F]DCFPyL by Ga- and Lu-complexed PSMA-targeted ligands.

PSMA binding affinity measurements were conducted by *in vitro* competition binding assays using PSMA-expressing LNCaP prostate cancer cells and [^18^F]DCFPyL as the radioligand. As shown in Figure 3, all tested ligands inhibited the binding of [^18^F]DCFPyL to LNCaP cells in a concentration-dependent manner. The calculated K_i_ values for Ga-HTK03177, Ga-HTK03187, Lu-HTK03170, Lu-HTK04048 and Lu-HTK04028 were 5.0±2.4, 10.6±2.0, 1.6±0.4, 1.4±1.0 and 13.9±3.2 nM, respectively.

### PET imaging and *ex vivo* biodistribution of ^68^Ga-labeled ligands

**Figure 4:**
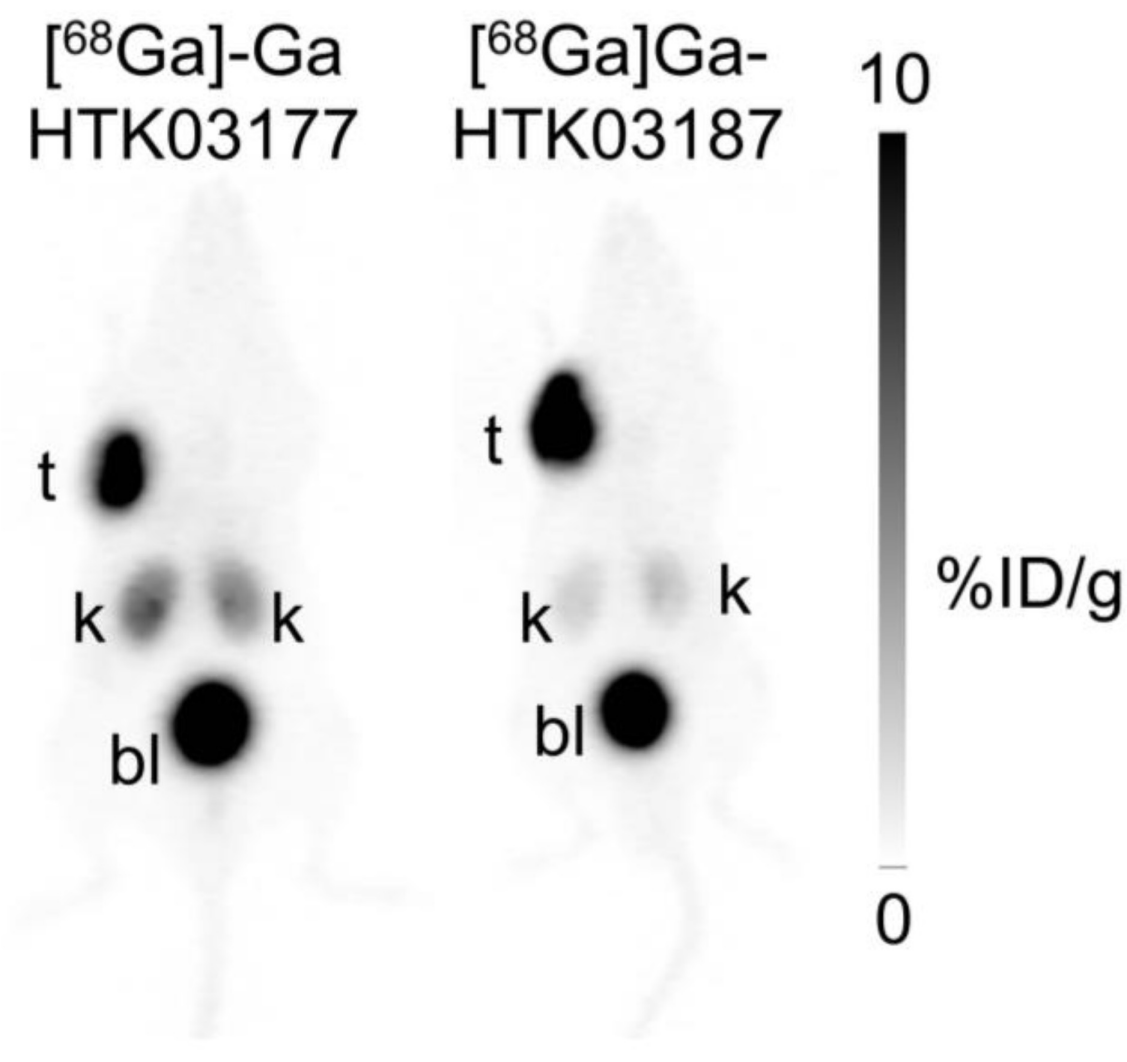
Representative maximum intensity projection PET images of [^68^Ga]Ga-HTK03177 and [^68^Ga]Ga-HTK03187 acquired at 1 h post-injection from LNCaP tumor-bearing mice.

PET imaging studies were conducted in mice bearing PSMA-expressing LNCaP tumor xenografts. As shown in Figure 4, both [^68^Ga]Ga-HTK03177 and [^68^Ga]Ga-HTK03187 clearly delineated LNCaP tumor xenografts in PET images with excellent tumor-to-background contrast. While both tracers had comparable high tumor uptake, the kidney uptake of [^68^Ga]Ga-HTK03177 was moderate, and the kidney uptake of [^68^Ga]Ga-HTK03187 was relatively lower. The uptake of both tracers in other normal organs/tissues was negligible and both tracers were excreted mainly via the renal pathway as high radioactivity accumulation in urinary bladder was clearly visualized in PET images.

**Figure 5:**
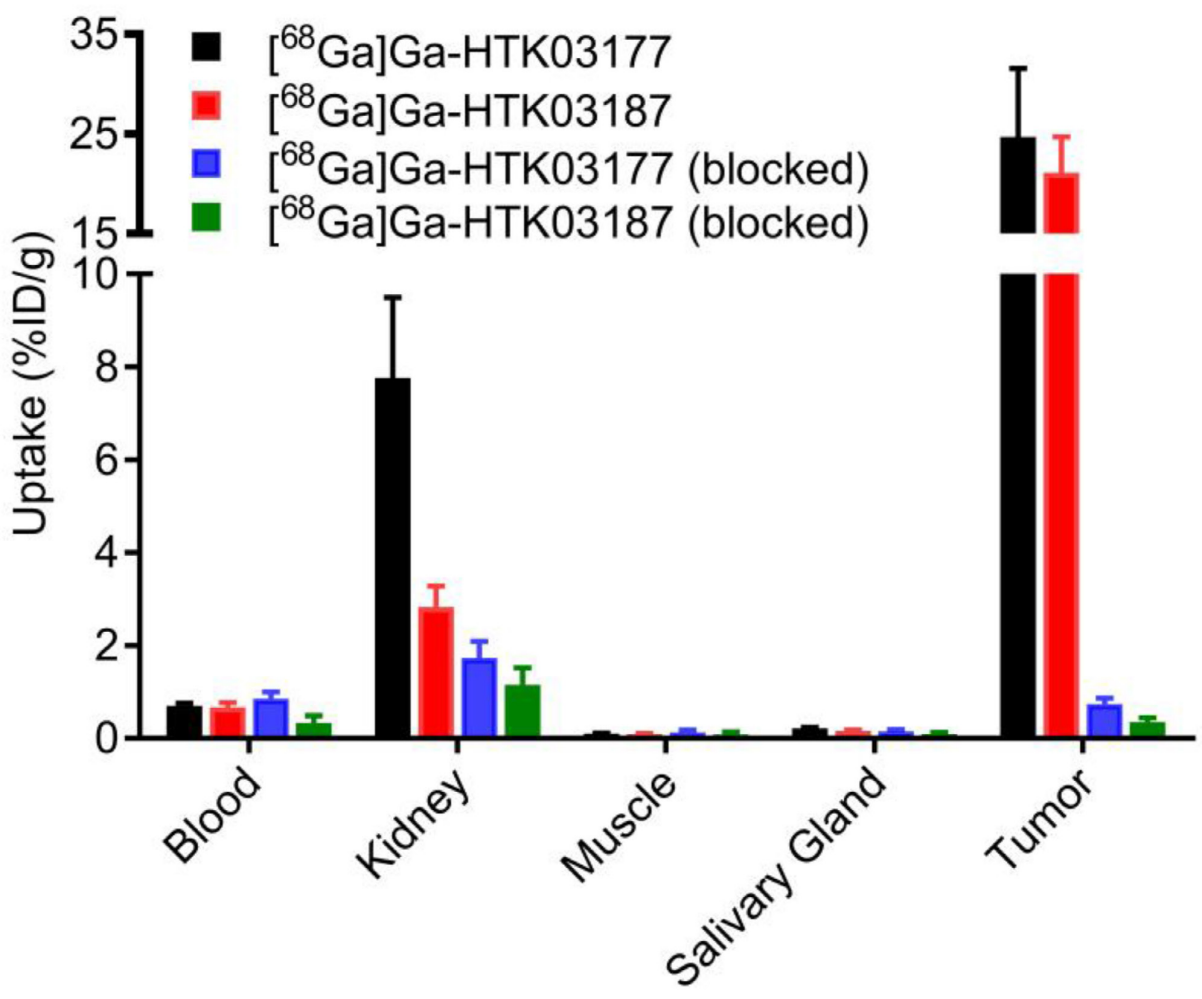
Uptake of ^68^Ga-labeled HTK03177 and HTK03187 in tumor and representative organs/tissues of LNCaP tumor-bearing mice collected at 1 h post-injection. The mice in the blocked groups were co-injected with 0.5 mg of DCFPyL.

The *ex vivo* biodistribution data of [^68^Ga]Ga-HTK03177 and [^68^Ga]Ga-HTK03187 (Figure 5 and Table S4) are consistent with the observations from their PET images (Figure 4). The tumor uptake values of [^68^Ga]Ga-HTK03177 and [^68^Ga]Ga-HTK03187 were 24.7±6.85 and 21.1±3.62 %ID/g, respectively. The kidney uptake values of [^68^Ga]Ga-HTK03177 and [^68^Ga]Ga-HTK03187 were 7.76±1.00 and 2.83±0.45 %ID/g, respectively. The relatively lower kidney uptake of [^68^Ga]Ga-HTK03187 compared with [^68^Ga]Ga-HTK03177 could be due to a relatively weaker PSMA binding affinity of [^68^Ga]Ga-HTK03187 vs [^68^Ga]Ga-HTK03177 (K_i_ = 10.6±2.0 vs 5.0±2.4 nM). The average uptake values for all other collected organs/tissues were ≤ 0.7 %ID/g for both tracers including salivary glands (0.22 %ID/g for [^68^Ga]Ga-HTK03177 and 0.16 %ID/g for [^68^Ga]Ga-HTK03187). The tumor-to-blood, tumor-to-muscle, tumor-to-kidney and tumor-to-salivary gland uptake ratios were 36.1±12.5, 265±103, 3.25±1.16 and 112±33.1, respectively for [^68^Ga]Ga-HTK03177, and 32.2±8.53, 249±61.2, 7.67±2.10 and 133±14.0, respectively for [^68^Ga]Ga-HTK03187. Co-injection of DCFPyL (0.5 mg) reduced the average uptake of LNCaP tumor xenografts and kidneys by 97 and 78%, respectively for [^68^Ga]Ga-HTK03177, and by 98 and 59%, respectively for [^68^Ga]Ga-HTK03187.

### SPECT imaging and *ex vivo* biodistribution of ^177^Lu-labeled ligands

**Figure 6:**
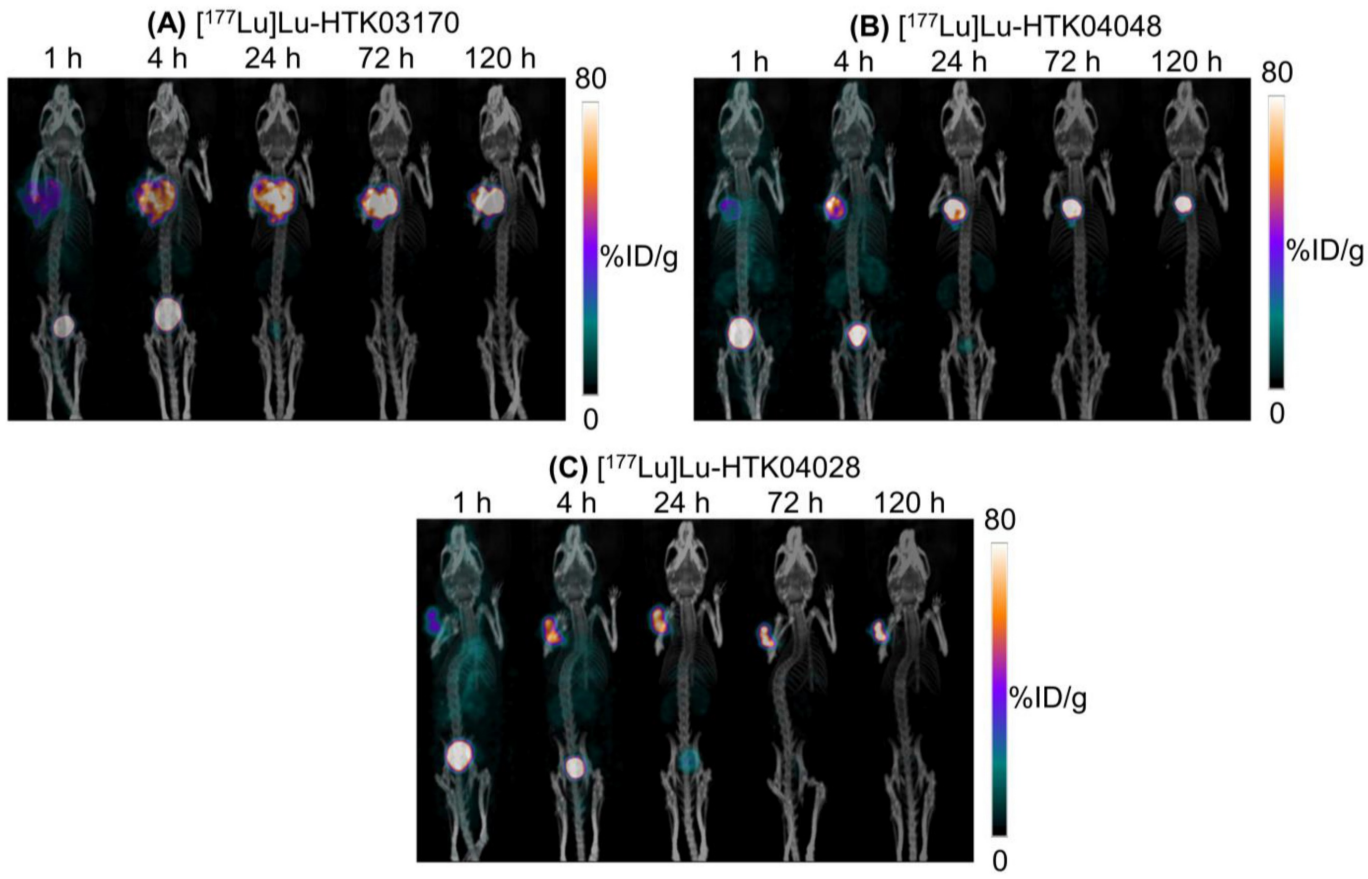
Representative longitudinal SPECT/CT images of (A) [^177^Lu]Lu-HTK03170, (B) [177Lu]Lu-HTK040 [177Lu]Lu-HTK04028" to "(B) [177Lu]Lu-HTK04048 and (C) [177Lu]Lu-HTK04028 acquired from LNCaP tumor-bearing mice.

The longitudinal SPECT/CT images of [^177^Lu]Lu-HTK03170, [^177^Lu]Lu-HTK04048 and [^177^Lu]Lu-HTK04028 are shown in Figure 6. A higher background uptake level was observed at the earlier time points (1 and 4 h post-injection) and the radioligands were excreted mainly via the renal pathway. The tumor uptake of all three radioligands peaked at 4-24 h post-injection, and the tumor uptake was relatively sustained over time. There was minimal uptake of all three radioligands in all normal organs/tissues including kidneys and salivary glands at 24 h post-injection and beyond. The tumor shrank from Day 1 to Day 5 indicates observable radiotherapeutic effect from the injection of ~37 MBq of [^177^Lu]Lu-HTK03170, [^177^Lu]Lu-HTK04048 and [^177^Lu]Lu-HTK04028.

**Figure 7:**
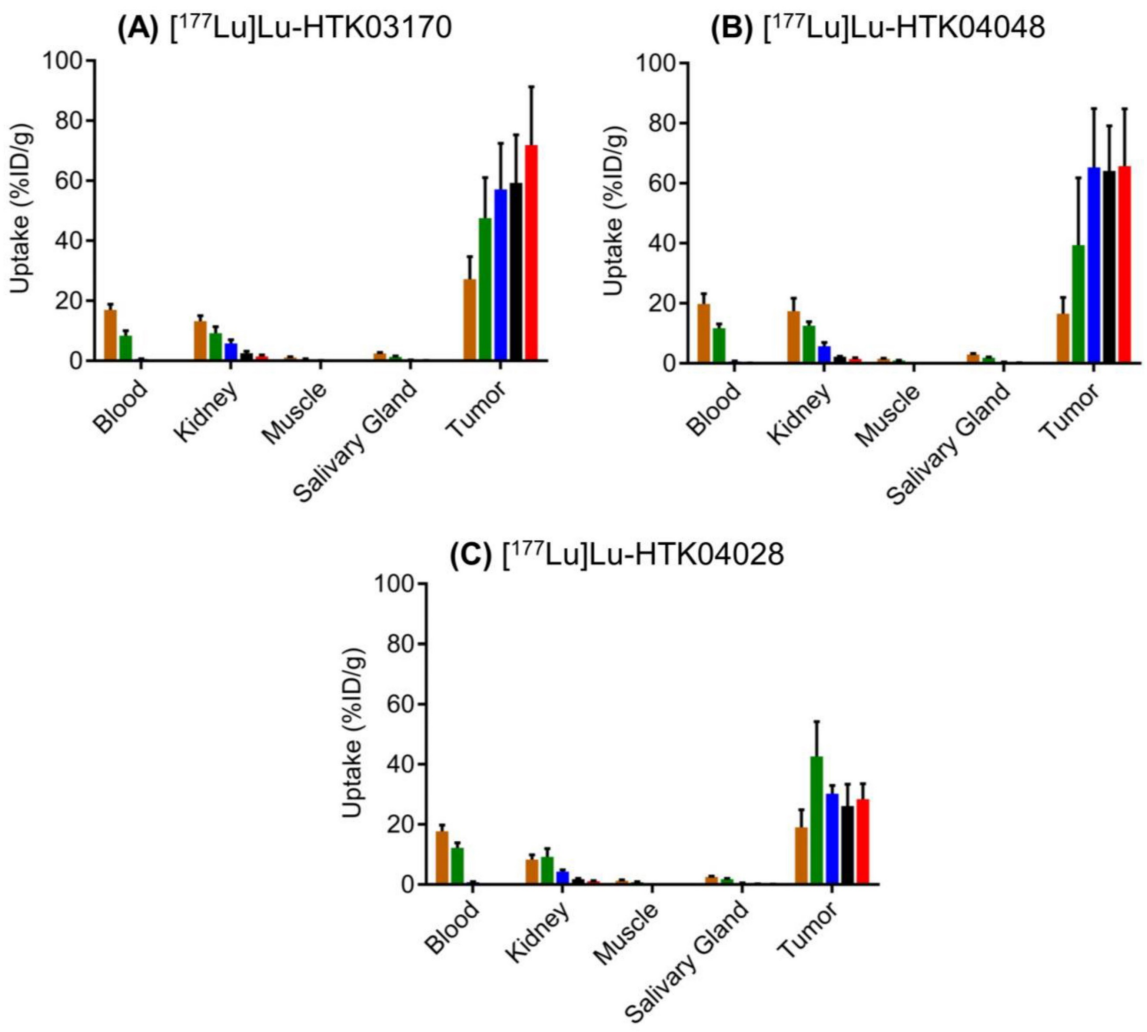
*Ex vivo* biodistribution of (A) ^177^Lu-HTK03170, (B) ^177^Lu-HTK04048 and (C) ^177^Lu-HTK04028 in mice bearing LNCaP tumor xenografts. Time points after injection: brown bar, 1 h; green bar, 4 h; blue bar, 24 h; black bar, 72 h; red bar, 120 h.

The *ex vivo* biodistribution data of [^177^Lu]Lu-HTK03170, [^177^Lu]Lu-HTK04048 and [^177^Lu]Lu-HTK04028 in LNCaP tumor-bearing mice (Figure 7 and Table S5-S7) are consistent with the observations from their SPECT/CT images (Figure 6). The average blood uptake of all three ^177^Lu-labeled ligands was ~ 17-20 %ID/g at 1 h post-injection, and reduced quickly over time. At 24 h post-injection, the average blood uptake of all three ^177^Lu-labeled ligands was ≤ 0.82 %ID/g. The average tumor uptake of both [^177^Lu]Lu-HTK03170 and [^177^Lu]Lu-HTK04048 peaked at 24 h post-injection, reached at ~60 %ID/g, and was sustained over time. The average tumor uptake of [^177^Lu]Lu-HTK04028 maximized at 4 h post-injection at ~43 %ID/g, and was reduced slowly to ~28 %ID/g at 120 h post-injection. The average kidney uptake of all three ^177^Lu-labeled ligands was moderate at 8.3 - 17.4 %ID/g at 1 h post-injection, reduced to 4.32- 5.80 %ID/g at 24 h post-injection and then 1.16-1.48 %ID/g at 120 h post-injection. The average salivary gland uptake of all three ^177^Lu-labeled ligands was low at 2.43-2.92 %ID/g at 1 h post-injection, and reduced continuously over time to 0.05 - 0.13 %ID/g at 120 h post-injection. The average uptake of all three ^177^Lu-labeled ligands in other collected organs/tissues was low (≤ 1.08 %ID/g) at 24 h post-injection and reduced continuously over time as well.

### Dosimetry and radioligand therapy studies

The absorbed doses of [^177^Lu]Lu-HTK03170 in LNCaP tumor and major mouse organs/tissues were calculated using the OLINDA software, and the results were compared with those of previously published [^177^Lu]Lu-HTK03149 and [^177^Lu]Lu-PSMA-617 12. As shown in Table S8, the radiation dose of [^177^Lu]Lu-HTK03170 delivered to LNCaP tumor xenografts was 6.7-fold and 16.4-fold of those previously reported for [^177^Lu]Lu-HTK03149 and [^177^Lu]Lu-PSMA-617, respectively. The radiation doses of [^177^Lu]Lu-HTK03170 calculated for the major organs of 25-g mice are shown in Table S9. Compared with [^177^Lu]Lu-HTK03149 and [^177^Lu]Lu-PSMA-617, [^177^Lu]Lu-HTK03170 delivered 1.4- to 8.4-fold and 1.4- to 26.5-fold to various organs, respectively. For the critical organ, kidneys, [^177^Lu]Lu-HTK03170 delivered 5.6- and 1.7-fold radiation dose when compared with those of [^177^Lu]Lu-HTK03149 and [^177^Lu]Lu-PSMA-617, respectively.

**Figure 8:**
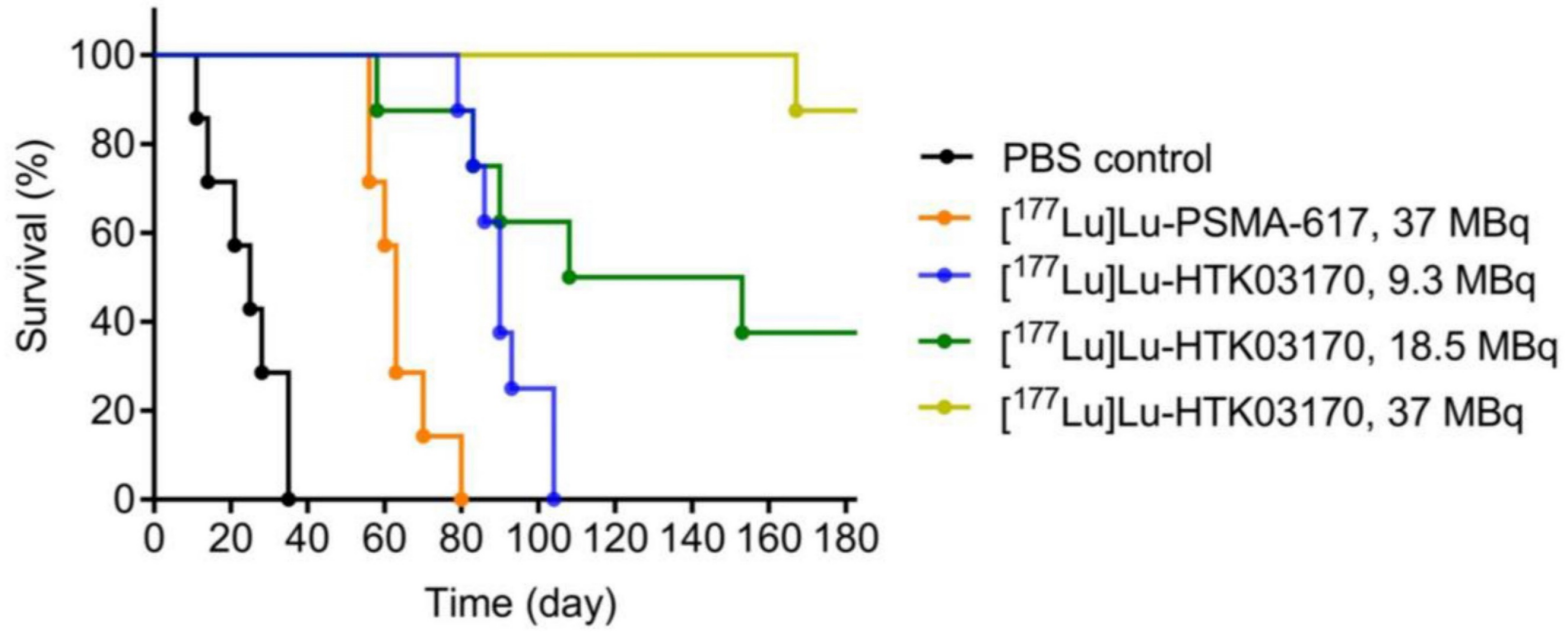
Survivals of mice treated with PBS, [^177^Lu]Lu-PSMA-617 (37 MBq) and various injected radioactivities (9.3 - 37 MBq) of ^177^Lu-HTK03170 (n = 7 - 8 per treatment group).

The radioligand therapy study of [^177^Lu]Lu-HTK03170 was conducted in LNCaP tumor-bearding mice uisng PBS as the control and [^177^Lu]Lu-PSMA-617 for comparison. As shown in Figure 8, the mice injected with PBS had a short median survival of only 25 days. The mice injected with 37 MBq of [^177^Lu]Lu-PSMA-617 had an extend median survival of 63 days. The median survivals for the groups of mice treated with [^177^Lu]Lu-HTK03170 depends on the injected radioactivity and a higher injected radioactivity led to a longer median survival. The meadian survivals for the mice treated with 9.3, 18.5 and 37 MBq of [^177^Lu]Lu-HTK03170 were 90, 131 and > 180 days, respectively. No toxicity was observed for all the treatment groups as no signifant weight loss was observed for all the treated mice (Figure S1-S5).

## Discussion

Replacing Glu in the PSMA-targeting pharmacophore with a different moiety for the design of novel radioligands have also been attempted by others. Felber et al. reported the replacement of Glu with various moieties including (S)-2-aminoheptanoic acid, (S)-2-amino-3-(furan-2-yl)propanoic acid, (S)-2-aminopent-4-ynoic acid and (S)-2-amino-4-(1H-1,2,3,4-tetrazol-5-yl)butanoic acid 19. However, such replacements led to significant reduction in PSMA binding affinity. Yang and Duan replaced Glu with (S)-2-amino-3-(carboxyformamido)propanoic acid for the design of PSMA-targeted agents 20-22. Despite preserved PSMA binding affinity, the ^68^Ga-labeled analogs derived from this new pharmacophore showed comparable or even higher kidney uptake than [^68^Ga]Ga-PSMA-617 20-21. Comparing our *in vivo* data with these reported data 20-21 is difficult due to the use of different tumor model (LNCaP vs 22Rv1) and mouse strain (NSG/NRG vs BALB/c nude). Their top candidate, [^68^Ga]Ga-P137, which showed a higher tumor uptake than [^68^Ga]Ga-PSMA-617 (6.43±0.98 vs 3.41±1.31 %ID/g at 1 h post-injection) in the preclinical tumor model was further evaluated in 3 prostate cancer patients and compared head-to-head with [^68^Ga]Ga-PSMA-617 21. The uptake patterns of [^68^Ga]Ga-P137 and [^68^Ga]Ga-PSMA-617 in prostate cancer lesions and normal human organs/tissues were comparable. However, [^68^Ga]Ga-P137 showed a much lower radioactivity accumulation in the urinary bladder, leading to a higher sensitivity for detecting intra-prostatic cancer lesions. These data suggest that [^68^Ga]Ga-P137 is a promising PSMA-targeted tracer for detecting prostate cancer lesions with PET and the reported novel PSMA-targeting pharmacophore, Lys-urea-(S)-2-amino-3-(carboxyformamido)propanoic acid is also promising for the design of PSMA-targeted radiotherapeutic agents labeled with β- or α-emitter such as ^177^Lu and ^225^Ac.

Recently we observed that treating mice with a high dose (up to 657 mg/kg) of monosodium glutamate reduced the uptake of [^68^Ga]Ga-PSMA-11 in mouse kidneys and salivary glands, but had no effect on the uptake in LNCaP tumor xenografts 23. This suggests that part of the high uptake of [^68^Ga]Ga-PSMA-11 in mouse kidneys and salivary glands might be mediated by off-targets other than PSMA, and the off-target uptake might be associated with the Glu at the Lys-urea-Glu pharmacophore of [^68^Ga]Ga-PSMA-11. This observation prompted us to investigate the effect of replacing Glu with a close analog (Asp, Aad and 2-aminopimelic acid (Api)) on the PSMA targeting capability and off-target uptake in kidneys and salivary glands 12. While the replacement with Api abolished the PSMA binding affinity, the Asp and Aad derivatives preserved PSMA binding affinity. Most importantly, compared with the Glu-containing [^68^Ga]Ga-HTK03041, the Aad-derived [^68^Ga]Ga-HTK03149 (Figure 2A, X = CH_2_) showed not only comparable uptake in PSMA-expressing LNCaP tumor xenografts (23.1 ± 6.11 vs 19.1 ± 6.37 %ID/g, 1 h post-injection), but also > 95% reduction in kidney (170±26.4 vs 4.15±1.46 %ID/g, 1 h post-injection) and salivary gland (4.99±0.88 vs 0.22 ± 0.06 %ID/g, 1 h post-injection) uptake 11-12.

In this study, we further investigated the effect of replacing Aad in [^68^Ga]Ga-HTK03149 with a close analog, Cmc ([^68^Ga]Ga-HTK03177, X = S, Figure 2A) and Cms ([^68^Ga]Ga-HTK03187, X = O, Figure 2A) on the PSMA targeting capability and off-target uptake. *In vitro* competition binding assays showed that the replacement was tolerable as the PSMA binding affinities (K_i_) of Ga-HTK03177 (5.0±2.4 nM) and Ga-HTK03187 (10.6±2.0 nM) were comparable to that of Ga-HTK03149 (6.99±0.80 nM) 12. Similarly, PET imaging and biodistribution studies of [^68^Ga]Ga-HTK03177 and [^68^Ga]Ga-HTK03187 in LNCaP tumor-bearing mice at 1 h post-injection also showed comparable patterns as previously observed using [^68^Ga]Ga-HTK03149 with high tumor uptake (~21-25 %ID/g) and low uptake in normal organs/tissues including kidneys (< 8.0 %ID/g) and salivary glands (< 0.25 %ID/g). This led to excellent tumor-to-background contrast PET images of [^68^Ga]Ga-HTK03177 and [^68^Ga]Ga-HTK03187, and supports their clinical translation for detecting prostate cancer lesions. Co-injection of DCFPyL (0.5 mg) reduced the average tumor uptake of [^68^Ga]Ga-HTK03177 and [^68^Ga]Ga-HTK03187 by ≥ 97%, further confirming the specific uptake of both tracers in tumors.

Subsequently we synthesized and evaluated three ^177^Lu-labeled DOTA-conjugated PSMA-targeted ligands based on these novel pharmacophores including Lys-urea-Aad-derived [^177^Lu]Lu-HTK03170, Lys-urea-Cmc-derived [^177^Lu]Lu-HTK04048 and Lys-urea-Cms-derived [^177^Lu]Lu-HTK04028 (Figure 2B). All these three radioligands were conjugated with a weak albumin binder, 4-(*p*-methoxyphenyl)butyric acid. Conjugating an albumin-binder is a common strategy for the design of radiotherapeutic ligands to extend their blood residence time and to enhance overall tumor uptake. This strategy has been widely applied to the design of radiotherapeutic agents targeting various cancer markers including PSMA 11,15,24-25. The most popular albumin binders are derivatives of Evans blue and 4-(*p*-iodophenyl)butyric acid 15,24-25. However, both Evans blue and 4-(*p*-iodophenyl)butyric acid have high binding affinity for albumin and their derived radiotherapeutic agents tend to have a long blood residence time, leading to a high undesired radiation absorbed dose to normal organs/tissues including bone marrow 15,24-25. To balance the overall tumor uptake and blood residence time of radiotherapeutic agents, we previously conducted structure-activity-relationship study on 4-(*p*-iodophenyl)butyric acid derivatives by replacing the *p*-iodo with various substituents 11. Among the evaluated analogs, 4-(*p*-methoxyphenyl)butyric acid was identified as the top candidate. The 4-(*p*-methoxyphenyl)butyric acid-conjugated [^177^Lu]Lu-HTK03123 achieved ~3-fold peaked tumor uptake compared with the non-conjugated [^68^Ga]Ga-HTK03041 (70.8 vs 23.1 %ID/g) and a relatively shorter blood residence time (< 1 %ID/g in blood at 24 h post-injection) 11.

*In vitro* competition binding assays showed that Lu-HTK03170, Lu-HTK04048 and Lu-HTK04028 retained high binding affinity to PSMA. This suggests that replacing Ga in Ga-HTK03149, Ga-HTK03177 and Ga-HTK03187 with Lu and the conjugation of 4-(*p*-methoxyphenyl)butyric acid as an albumin binder did not interfere their binding to PSMA. SPECT/CT imaging and *ex vivo* biodistribution studies also showed low kidney and salivary gland uptake of these ^177^Lu-labeled radioligands. These results were consistent with the observations from using ^68^Ga-labeled HTK03149 12, HTK03177 and HTK03187, suggesting replacing Ga in Ga-HTK03149, Ga-HTK03177 and Ga-HTK03187 with Lu and the conjugation of an albumin binder did not affect their good selectivity of PSMA to other off-targets either. The blood uptake of all ^177^Lu-labeled radioligands was ~17-20 %ID/g at 1 h post-injection and dropped to < 1 %ID/g at 24 h post-injection, which was consistent with our previous observations from using the 4-(*p*-methoxyphenyl)butyric acid-conjugated [^177^Lu]Lu-HTK03123, further confirming 4-(*p*-methoxyphenyl)butyric acid is a moderate albumin binder. Compared with [^68^Ga]HTK03149 12 and [^68^Ga]HTK03187, their ^177^Lu-labeled 4-(*p*-methoxyphenyl)butyric acid-conjugated analogs ([^177^Lu]Lu-HTK03170 and [^177^Lu]Lu-HTK34048, respectively) also showed ~3-fold peaked tumor uptake (~20 vs ~60 %ID/g). Unlike the high and sustained tumor uptake of [^177^Lu]Lu-HTK03170 and [^177^Lu]Lu-HTK34048, the peaked tumor uptake of [^177^Lu]Lu-HTK04028 was lower (42.6 %ID/g at 4 h post-injection) and the tumor uptake was reduced slowly afterwards. This is likely due to its relatively lower PSMA binding affinity (K_i_ = 13.9 nM) compared to those of Lu-HTK03170 (1.6 nM) and Lu-HTK04048 (1.4 nM).

Based on the *ex vivo* biodistribution data, [^177^Lu]Lu-HTK03170 was selected as our lead candidate for further development. Although [^177^Lu]Lu-HTK04048 performed equally well, its thioether moiety in Cmc could be oxidized to sulfoxide, potentially leading to instability in final drug formulation and a relatively shorter shelf life. Dosimetry calculation for [^177^Lu]Lu-HTK03170 was conducted and the results were compared with those previously obtained for [^177^Lu]Lu-HTK03149 and [^177^Lu]Lu-PSMA-617 (Table S8-S9). As expected, the albumin-binder-conjugated [^177^Lu]Lu-HTK03170 delivered a higher radiation dose (6.7-fold) to tumor than its non-albumin-binder-conjugated analog [^177^Lu]Lu-HTK03149. Most importantly, compared with the 6.7-fold tumor absorbed dose, its kidney absorbed dose was only 5.6-fold compared to that of [^177^Lu]Lu-HTK03149, leading to a better tumor-to-kidney absorbed dose ratio for [^177^Lu]Lu-HTK03170. Similarly, compared with the US FDA-approved [177Lu]PSMA-617" to "[177Lu]Lu-PSMA-617, [^177^Lu]Lu-HTK03170 delivered 16.4-fold tumor absorbed dose, but only 1.7-fold absorbed dose to kidneys. The superior tumor-to-kidney absorbed dose ratio for [^177^Lu]Lu-HTK03170 compared to that of [^177^Lu]Lu-PSMA-617 is mainly due to the use of the more PSMA-selective Lys-urea-Aad pharmacophore compared with the common Lys-urea-Glu pharmacophore in [^177^Lu]Lu-PSMA-617. This suggests that only a fraction of the standard clinical injected radioactivity (7.4 GBq) of [^177^Lu]Lu-PSMA-617 is needed for [^177^Lu]Lu-HTK03170 to achieve comparable or even better treatment efficacy, and the use of [^177^Lu]Lu-HTK03170 is less likely to cause nephrotoxicity. However, it should be noted that compared to [^177^Lu]Lu-PSMA-617, although [^177^Lu]Lu-HTK03170 has 16.4-fold tumor absorbed dose, it also has 23.4-fold absorbed dose to skeleton which is used as a surrogate for bone marrow. Therefore, the tumor-to-bone marrow absorbed dose ratio of [^177^Lu]Lu-HTK03170 is actually inferior to that of [^177^Lu]Lu-PSMA-617, and potential bone marrow toxicity should be closely monitored when translating [^177^Lu]Lu-HTK03170 to the clinic.

Radioligand therapy studies confirmed the results from dosimetry calculation as 9.3 MBq of [^177^Lu]Lu-HTK03170 led to a longer median survival than 37 MBq of [^177^Lu]Lu-PSMA-617 (90 vs 63 days, Figure 8). We also raised the injected radioactivity of [^177^Lu]Lu-HTK03170 to 37 MBq. The resulting excellent median survival (> 180 days) along with the observed continuously increasing body weight of the treated mice (Figure S5) demonstrate that [^177^Lu]Lu-HTK03170 is an effective and safe radiotherapeutic agent for the treatment of metastatic prostate cancer.

It should be noted that although the high uptake of reported PSMA-targeted radioligands in kidneys and salivary glands has been suggested to be mediated by off-targets other than PSMA 26-28, the identities of these off-targets have not been confirmed and remain controversial 28-29. We observed low uptake of radiolabeled ligands derived from Lys-urea-Aad, Lys-urea-Cmc and Lys-urea-Cms pharmacophores in mouse kidneys and salivary glands. However, the species difference between human PSMA expressed in LNCaP tumor xenografts and mouse PSMA expressed in mouse kidneys and salivary glands cannot be ruled out. It has been reported that mouse PSMA is more selective for Glu-containing ligands whereas human PSMA is more tolerable for replacing the Glu with other close analogs 30. Whether the low kidney and salivary gland uptake of radioligands derived from these novel PSMA-targeting pharmacophores in mice could be translated in humans remains to be investigated. We are currently conducting a phase I/II clinical trial to treat metastatic castration-resistant prostate cancer with [^177^Lu]Lu-HTK03170 31. The preliminary toxicity, dosimetry, biodistribution and treatment efficacy of [^177^Lu]Lu-HTK03170 will be reported at the conclusion of the clinical trial.

## Conclusions

Aad in the previously reported [^68^Ga]Ga-HTK03149 could be replaced with a close analog, Cmc and Cms, and the resulting Lys-urea-Cmc ([^68^Ga]Ga-HTK03177) and Lys-urea-Cms ([^68^Ga]Ga-HTK03187) derivatives retain excellent PSMA-targeting capability and low uptake in common off-target organs including kidneys and salivary glands. ^177^Lu-labeled albumin-binder-conjugated Lys-urea-Aad, Lys-urea-Cmc and Lys-urea-Cms derivatives could further enhance uptake in PSMA-expressing tumor xenografts without significantly increasing uptake in kidneys and salivary glands. Lys-urea-Aad, Lys-urea-Cmc and Lys-urea-Cms are promising pharmacophores for the design of PSMA-targeted radioligands especially for the radiotherapeutic applications to minimize toxicity to kidneys and salivary glands.

## Supplementary Material

Supplementary figures and tables. Click here for additional data file. Results/Information for the synthesis and purification of DOTA-conjugated PSMA-targeted ligands (Table S1), their nonradioactive Ga- and Lu-complexed standards (Table S2), and ^68^Ga- and ^177^Lu-labeled analogs (Table S3), biodistribution data (Tables S4-S7), the results of radiation dosimetry calculations (Tables S8-S9), and the change of tumor volume and mouse body weight for the radioligand therapy studies (Figures S1-S5) are provided as supplementary material.

## Abbreviations

Aad: 2-aminoadipic acid
Api: 2-aminopimelic acid
Asp: aspartic acid
ATCC: American Type Culture Collection
CH_2_Cl_2_: dichloromethane
Cmc: *S*-carboxylmethylcysteine
Cms: *O*-carboxylmethylserine
CT: computed tomography
DIEA: diisopropylethylamine
DMF: *N*,*N*-dimethylformamide
DOTA: 1,4,7,10-tetraazacyclododecane-1,4,7,10-tetraacetic acid
FDA: Food and Drug Administration
FmocOSu: *N*-(9H-fluoren-9-ylmethoxycarbonyloxy)succinimide
GaCl_3_: gallium(III) chloride
Glu: glutamic acid
HATU: hexafluorophosphate azabenzotriazole tetramethyl uronium
HCl: hydrogen chloride
HEPES: 2-[4-(2-hydroxyethyl)piperazin-1-yl]ethanesulfonic acid
HPLC: high performance liquid chromatography
LuCl_3_: lutetium(III) chloride
Lys: lysine
MgSO_4_: magnesium sulfate
NaN_3_: sodium azide
NaOAc: sodium acetate
PBS: phosphate-buffered saline
PET: positron emission tomography
PSMA: prostate-specific membrane antigen
RPMI: Roswell Park Memorial Institute
SPECT: single photon emission computed tomography
%ID/g: percent injected dose per gram of tissue
