# Supplementary material for: Lys-urea-Aad, Lys-urea-Cmc and Lys-urea-Cms as potential pharmacophores for the design of PSMA-targeted radioligands to reduce off-target uptake in kidneys and salivary glands
Source: Theranostics. 2023 Aug 15;13(13):4559–73. doi: 10.7150/thno.87663 (PMC10465233; doi:10.7150/thno.87663)
Supplement: Supplementary file 1 — Supplementary figures and tables. [file thnov13p4559s1.pdf]

SUPPLEMENTARY MATERIAL

Lys-urea-Aad, Lys-urea-Cmc and Lys-urea-Cms as potential pharmacophores for the design of PSMA-targeted radioligands to reduce off-target uptake in kidneys and salivary glands

Hsiou-Ting Kuo[1], Zhengxing Zhang[1], Chengcheng Zhang[1], Helen Merkens[1], Ruiyan Tan[1], Antonio A. W. L. Wong[1], Carlos F. Uribe[2,3], François Bénard[1,2,3,*], Kuo-Shyan Lin[1,2,3,*]

[1]Department of Molecular Oncology, BC Cancer, Vancouver, BC V5Z1L3, Canada
[2]Department of Functional Imaging, BC Cancer, Vancouver, BC V5Z4E6, Canada
[3]Department of Radiology, University of British Columbia, Vancouver, BC V5Z1M9, Canada

Corresponding Authors:

*François Bénard, MD
Address: 675 West 10th Avenue, Rm 14-111, Vancouver, BC V5Z1L3, Canada
Phone: 604-675-8206
Fax: 604-675-8218
E-mail: fbenard@bccrc.ca

*Kuo-Shyan Lin, PhD
Address: 675 West 10th Avenue, Rm 4-123, Vancouver, BC V5Z1L3, Canada
Phone: 604-675-8208
Fax: 604-675-8218
E-mail: klin@bccrc.ca

Table S1: HPLC purification conditions and MS characterizations of DOTA-conjugated PSMA-targeted ligands.

Compound name	HPLC conditions	Retention time (min)	Yield (%)	Calculated mass (m/z)	Found (m/z)
HTK03177	System A/Column A: 24% CH_3CN and 0.1% TFA in H_2O; flow rate 30 mL/min	8.2	34	$[M+H]^+$ 1124.5	$[M+H]^+$ 1124.5
HTK03187	System B/Column B: 28% CH_3CN and 0.1% TFA in H_2O; flow rate 4.5 mL/min	10.4	30	$[M+H]^+$ 1108.5	$[M+H]^+$ 1108.5
HTK03170	System B/Column B: 34% CH_3CN and 0.1% TFA in H_2O; flow rate 4.5 mL/min	13.1	4	$[M+2H]^{2+}$ 734.4	$[M+2H]^{2+}$ 734.6
HTK04048	System B/Column B: 35% CH_3CN and 0.1% TFA in H_2O; flow rate 4.5 mL/min	11.7	53	$[M+H]^+$ 1485.7	$[M+H]^+$ 1486.0
HTK04028	System A/Column A: 28% CH_3CN and 0.1% TFA in H_2O; flow rate 30 mL/min	14.8	21	$[M+H]^+$ 1469.7	$[M+H]^+$ 1469.7

Table S2: HPLC purification conditions and MS characterizations of nonradioactive Ga- and Lu-complexed PSMA-targeted ligands.

Compound name	HPLC conditions	Retention time (min)	Yield (%)	Calculated mass (m/z)	Found (m/z)
Ga-HTK03177	System B/Column B: 32% CH_3CN and 0.1% TFA in H_2O; flow rate 4.5 mL/min	7.8	56	$[M+H]^+$ 1190.4	$[M+H]^+$ 1190.3
Ga-HTK03187	System B/Column B: 29% CH_3CN and 0.1% TFA in H_2O; flow rate 4.5 mL/min	13.3	53	$[M+H]^+$ 1174.4	$[M+H]^+$ 1174.3
Lu-HTK03170	System B/Column B: 34% CH_3CN and 0.1% TFA in H_2O; flow rate 4.5 mL/min	15.4	95	$[M+H]^+$ 1639.7	$[M+H]^+$ 1639.7
Lu-HTK04048	System B/Column B: 35% CH_3CN and 0.1% TFA in H_2O; flow rate 4.5 mL/min	13.9	70	$[M+H]^+$ 1657.6	$[M+H]^+$ 1657.9
Lu-HTK04028	System B/Column B: 35% CH_3CN and 0.1% TFA in H_2O; flow rate 4.5 mL/min	11.5	29	$[M+H]^+$ 1641.6	$[M+H]^+$ 1641.8

Table S3: HPLC conditions for the purification and quality control of ^{68}Ga- and ^{177}Lu-labeled PSMA-targeted ligands.

Compound name	HPLC conditions		Retention time (min)
[^{68}Ga]Ga-HTK03177	Semi-prep	System C/Column B: 31% CH_3CN and 0.1% TFA in H_2O; flow rate 4.5 mL/min	12.5
	QC	System C/Column C: 32% CH_3CN and 0.1% TFA in H_2O; flow rate 2.0 mL/min	6.0
[^{68}Ga]Ga-HTK03187	Semi-prep	System C/Column B: 29% CH_3CN and 0.1% TFA in H_2O; flow rate 4.5 mL/min	15.5
	QC	System C/Column C: 31% CH_3CN and 0.1% TFA in H_2O; flow rate 2.0 mL/min	6.1
[^{177}Lu]Lu-HTK03170	Semi-prep	System C/Column B: 33% CH_3CN and 0.1% TFA in H_2O; flow rate 4.5 mL/min	23.9
	QC	System C/Column C: 34% CH_3CN and 0.1% TFA in H_2O; flow rate 2 mL/min	8.1
[^{177}Lu]Lu-HTK04048	Semi-prep	System C/Column B: 35% CH_3CN and 0.1% TFA in H_2O; flow rate 4.5 mL/min	18.4
	QC	System C/Column C: 37% CH_3CN and 0.1% TFA in H_2O; flow rate 2.0 mL/min	5.7
[^{177}Lu]Lu-HTK04028	Semi-prep	System C/Column B: 34% CH_3CN and 0.1% TFA in H_2O; flow rate 4.5 mL/min	18.7
	QC	System C/Column C: 35% CH_3CN and 0.1% TFA in H_2O; flow rate 2.0 mL/min	7.0

Table S4: Biodistribution (mean ± SD) and uptake ratios of ^{68}Ga-labeled PSMA-targeted tracers in LNCaP tumor-bearing mice. The mice in the blocked group were co-injected with DCFPyL (0.5 mg). Statistical analyses were conducted for the data of [^{68}Ga]Ga-HTK03177 and [^{68}Ga]Ga-HTK03187 with/without co-injection of DCFPyL(0.5 mg). *, $p < 0.05$; **, $p < 0.01$; ***, $p < 0.001$.

Tissue (%ID/g)	**[^{68}Ga]Ga-HTK03177**		**[^{68}Ga]Ga-HTK03187**	
	1 h (n = 5)	1 h blocked (n = 4)	1 h (n = 5)	1 h blocked (n = 4)
Blood	0.70 ± 0.06	0.85 ± 0.15*	0.67 ± 0.10	0.33 ± 0.16**
Fat	0.09 ± 0.04	0.20 ± 0.16	0.07 ± 0.02	0.06 ± 0.04
Testes	0.25 ± 0.07	0.36 ± 0.14	0.15 ± 0.03	0.15 ± 0.01
Intestines	0.21 ± 0.02	0.69 ± 0.76	0.23 ± 0.05	0.13 ± 0.03**
Stomach	0.15 ± 0.09	0.12 ± 0.05	0.08 ± 0.05	0.03 ± 0.01*
Spleen	0.42 ± 0.15	0.25 ± 0.18	0.17 ± 0.02	0.12 ± 0.03*
Liver	0.33 ± 0.20	0.37 ± 0.22	0.21 ± 0.05	0.16 ± 0.05
Pancreas	0.14 ± 0.01	0.54 ± 0.54	0.12 ± 0.02	0.07 ± 0.02**
Adrenal glands	0.46 ± 0.17	0.61 ± 0.38	0.26 ± 0.06	0.13 ± 0.06**
Kidneys	7.76 ± 1.00	1.73 ± 0.36***	2.83 ± 0.45	1.15 ± 0.37***
Lungs	0.61 ± 0.08	0.54 ± 0.06	0.52 ± 0.09	0.31 ± 0.10**
Heart	0.21 ± 0.01	0.23 ± 0.04	0.19 ± 0.04	0.10 ± 0.05*
Tumor	24.7 ± 6.85	0.74 ± 0.12***	21.1 ± 3.62	0.35 ± 0.09***
Muscle	0.10 ± 0.01	0.13 ± 0.05	0.09 ± 0.01	0.08 ± 0.06
Bone	0.10 ± 0.03	0.24 ± 0.17	0.13 ± 0.03	0.06 ± 0.02**
Brain	0.02 ± 0.00	0.02 ± 0.00	0.02 ± 0.00	0.01 ± 0.00*
Thyroid	0.23 ± 0.01	0.21 ± 0.06	0.18 ± 0.02	0.12 ± 0.05*
Salivary glands	0.22 ± 0.02	0.15 ± 0.04**	0.16 ± 0.02	0.09 ± 0.04**
Lacrimal glands	0.12 ± 0.06	0.15 ± 0.10	0.09 ± 0.03	0.06 ± 0.03
Tumor:Blood	36.1 ± 12.5	0.89 ± 0.19***	32.2 ± 8.53	1.14 ± 0.25***
Tumor:Muscle	265 ± 103	5.50 ± 2.68*	249 ± 61.2	5.34 ± 2.25***
Tumor:Kidney	3.25 ± 1.16	0.43 ± 0.05**	7.67 ± 2.10	0.31 ± 0.08***
Tumor:Salivary gland	112 ± 33.1	5.05 ± 0.77***	133 ± 14.0	4.13 ± 0.92***
Blood:Salivary gland	3.14 ± 0.19	6.07 ± 2.53*	4.27 ± 0.74	3.64 ± 0.44

Table S5: Biodistribution (mean ± SD) and uptake ratios of [^{177}Lu]Lu-HTK03170 in LNCaP tumor-bearing mice.

Tissue (%ID/g)	1 h (n = 6)	4 h (n = 6)	24 h (n = 6)	72 h (n = 6)	120 h (n = 6)
Blood	16.9 ± 1.97	8.34 ± 1.67	0.59 ± 0.13	0.06 ± 0.02	0.02 ± 0.01
Fat	1.38 ± 0.45	0.60 ± 0.13	0.12 ± 0.04	0.04 ± 0.02	0.04 ± 0.03
Testes	2.17 ± 0.32	1.83 ± 0.58	0.64 ± 0.53	0.24 ± 0.04	0.16 ± 0.04
Intestines	1.19 ± 0.25	0.61 ± 0.17	0.18 ± 0.07	0.14 ± 0.11	0.05 ± 0.06
Stomach	0.59 ± 0.12	0.42 ± 0.15	0.25 ± 0.13	0.28 ± 0.33	0.08 ± 0.15
Spleen	1.64 ± 0.83	0.96 ± 0.29	0.45 ± 0.10	0.38 ± 0.15	0.53 ± 0.35
Liver	2.67 ± 0.37	1.89 ± 0.69	0.69 ± 0.27	0.36 ± 0.13	0.26 ± 0.11
Pancreas	1.41 ± 0.31	0.74 ± 0.18	0.12 ± 0.03	0.04 ± 0.01	0.02 ± 0.02
Adrenal glands	3.34 ± 0.45	1.78 ± 0.45	0.50 ± 0.16	0.27 ± 0.11	0.23 ± 0.18
Kidneys	13.2 ± 1.87	9.23 ± 2.18	5.80 ± 1.24	2.56 ± 0.62	1.48 ± 0.44
Lungs	8.06 ± 1.48	4.35 ± 0.82	0.72 ± 0.19	0.18 ± 0.04	0.07 ± 0.02
Heart	3.60 ± 0.52	1.91 ± 0.40	0.26 ± 0.04	0.09 ± 0.03	0.05 ± 0.02
Tumor	27.2 ± 7.55	47.6 ± 13.5	57.2 ± 15.3	59.3 ± 16.0	71.9 ± 19.4
Muscle	1.15 ± 0.19	0.58 ± 0.13	0.08 ± 0.02	0.02 ± 0.01	0.01 ± 0.01
Bone	0.89 ± 0.27	0.45 ± 0.13	0.06 ± 0.04	0.03 ± 0.02	0.02 ± 0.01
Brain	0.23 ± 0.04	0.13 ± 0.03	0.02 ± 0.01	0.01 ± 0.00	0.00 ± 0.00
Thyroid	2.90 ± 0.41	1.62 ± 0.42	0.37 ± 0.06	0.15 ± 0.01	0.09 ± 0.04
Salivary glands	2.43 ± 0.33	1.31 ± 0.30	0.28 ± 0.04	0.11 ± 0.03	0.05 ± 0.03
Lacrimal glands	0.27 ± 0.08	0.15 ± 0.07	0.04 ± 0.03	0.01 ± 0.01	0.00 ± 0.00
Tumor:Blood	1.58 ± 0.26	5.81 ± 1.58	105 ± 57.4	1029 ± 459	3996 ± 2182
Tumor:Muscle	23.5 ± 4.66	83.1 ± 17.0	736 ± 333	3374 ± 1417	12778 ± 19955
Tumor:Kidney	2.07 ± 0.55	5.38 ± 1.91	10.5 ± 4.77	24.0 ± 6.93	46.4 ± 21.3
Tumor:Salivary gland	11.3 ± 2.76	36.4 ± 5.35	202 ± 49.3	566 ± 236	1167 ± 531
Blood:Salivary gland	7.04 ± 0.86	6.48 ± 1.02	2.11 ± 0.47	0.57 ± 0.17	0.31 ± 0.06

Table S6: Biodistribution (mean ± SD) and uptake ratios of [^{177}Lu]Lu-HTK04048 in LNCaP tumor-bearing mice.

Tissue (%ID/g)	1 h (n = 7)	4 h (n = 7)	24 h (n = 7)	72 h (n = 7)	120 h (n = 8)
Blood	19.8 ± 3.38	11.7 ± 1.39	0.62 ± 0.16	0.08 ± 0.06	0.04 ± 0.03
Fat	1.56 ± 0.45	0.94 ± 0.13	0.15 ± 0.05	0.07 ± 0.03	0.05 ± 0.03
Testes	2.60 ± 0.62	2.72 ± 0.34	0.67 ± 0.24	0.35 ± 0.05	0.22 ± 0.09
Intestines	1.38 ± 0.22	1.00 ± 0.08	0.46 ± 0.31	0.13 ± 0.08	0.10 ± 0.10
Stomach	0.75 ± 0.11	0.71 ± 0.13	0.83 ± 0.66	0.20 ± 0.17	0.04 ± 0.02
Spleen	2.57 ± 0.48	1.96 ± 0.39	0.64 ± 0.10	0.54 ± 0.14	0.55 ± 0.27
Liver	3.94 ± 0.69	2.28 ± 0.46	0.77 ± 0.52	0.25 ± 0.02	0.18 ± 0.07
Pancreas	1.77 ± 0.17	1.15 ± 0.14	0.15 ± 0.03	0.06 ± 0.01	0.05 ± 0.03
Adrenal glands	4.43 ± 1.11	2.17 ± 0.21	0.67 ± 0.26	0.33 ± 0.06	0.30 ± 0.14
Kidneys	17.4 ± 4.32	12.5 ± 1.35	5.68 ± 1.28	2.19 ± 0.21	1.43 ± 0.48
Lungs	9.52 ± 1.44	5.92 ± 0.79	1.05 ± 0.36	0.26 ± 0.08	0.12 ± 0.05
Heart	4.74 ± 0.60	2.87 ± 0.30	0.34 ± 0.05	0.14 ± 0.03	0.08 ± 0.02
Tumor	16.5 ± 5.48	39.4 ± 22.4	65.3 ± 19.6	64.1 ± 15.0	65.7 ± 19.1
Muscle	1.54 ± 0.20	0.96 ± 0.09	0.11 ± 0.02	0.03 ± 0.01	0.02 ± 0.01
Bone	1.10 ± 0.16	0.69 ± 0.11	0.11 ± 0.02	0.06 ± 0.02	0.05 ± 0.02
Brain	0.29 ± 0.03	0.18 ± 0.01	0.03 ± 0.00	0.01 ± 0.00	0.01 ± 0.00
Thyroid	3.97 ± 0.47	2.68 ± 0.23	0.53 ± 0.07	0.28 ± 0.05	0.17 ± 0.02
Salivary glands	2.92 ± 0.36	1.91 ± 0.20	0.39 ± 0.08	0.18 ± 0.03	0.13 ± 0.03
Lacrimal glands	0.42 ± 0.12	0.54 ± 0.52	0.06 ± 0.01	0.01 ± 0.01	0.01 ± 0.01
Tumor:Blood	0.84 ± 0.22	3.49 ± 2.31	113 ± 44.5	1493 ± 1213	4346 ± 4168
Tumor:Muscle	10.7 ± 2.87	42.2 ± 27.3	594 ± 177	2043 ± 734	3358 ± 2122
Tumor:Kidney	0.99 ± 0.34	3.27 ± 2.16	11.5 ± 2.22	29.2 ± 6.22	52.1 ± 16.3
Tumor:Salivary gland	5.64 ± 1.51	20.9 ± 11.9	175 ± 58.1	362 ± 111	604 ± 226
Blood:Salivary gland	6.78 ± 0.76	6.12 ± 0.58	1.61 ± 0.40	0.43 ± 0.28	0.22 ± 0.13

Table S7: Biodistribution (mean ± SD) and uptake ratios of [^{177}Lu]Lu-HTK04028 in LNCaP tumor-bearing mice.

Tissue (%ID/g)	1 h (n = 5)	4 h (n = 5)	24 h (n = 5)	72 h (n = 5)	120 h (n = 6)
Blood	17.7 ± 2.10	12.2 ± 1.66	0.82 ± 0.11	0.05 ± 0.01	0.02 ± 0.00
Fat	1.26 ± 0.21	0.89 ± 0.24	0.14 ± 0.02	0.06 ± 0.02	0.03 ± 0.01
Testes	2.26 ± 0.46	2.66 ± 0.41	0.55 ± 0.05	0.31 ± 0.05	0.22 ± 0.01
Intestines	1.23 ± 0.24	1.04 ± 0.12	0.23 ± 0.05	0.07 ± 0.03	0.08 ± 0.05
Stomach	0.51 ± 0.18	0.45 ± 0.06	0.31 ± 0.10	0.07 ± 0.07	0.10 ± 0.10
Spleen	1.53 ± 0.23	1.44 ± 0.31	0.48 ± 0.14	0.49 ± 0.16	0.54 ± 0.15
Liver	2.93 ± 0.51	2.29 ± 0.55	0.54 ± 0.09	0.22 ± 0.02	0.16 ± 0.02
Pancreas	1.53 ± 0.22	1.24 ± 0.04	0.15 ± 0.02	0.05 ± 0.01	0.03 ± 0.00
Adrenal glands	3.25 ± 0.34	2.18 ± 0.33	0.73 ± 0.13	0.48 ± 0.17	0.33 ± 0.05
Kidneys	8.30 ± 1.58	9.21 ± 2.76	4.32 ± 0.58	1.77 ± 0.25	1.16 ± 0.13
Lungs	7.88 ± 1.77	5.79 ± 0.44	1.08 ± 0.72	0.18 ± 0.02	0.09 ± 0.01
Heart	4.15 ± 0.31	2.67 ± 0.31	0.36 ± 0.03	0.12 ± 0.03	0.07 ± 0.01
Tumor	19.0 ± 5.86	42.6 ± 11.6	30.2 ± 2.74	26.1 ± 7.29	28.4 ± 5.11
Muscle	1.32 ± 0.19	0.83 ± 0.09	0.11 ± 0.02	0.03 ± 0.01	0.02 ± 0.00
Bone	0.99 ± 0.21	0.63 ± 0.10	0.11 ± 0.02	0.05 ± 0.02	0.04 ± 0.02
Brain	0.26 ± 0.03	0.19 ± 0.02	0.02 ± 0.00	0.01 ± 0.00	0.00 ± 0.00
Thyroid	3.30 ± 0.42	2.30 ± 0.30	0.52 ± 0.08	0.24 ± 0.04	0.13 ± 0.02
Salivary glands	2.55 ± 0.21	1.75 ± 0.25	0.45 ± 0.10	0.19 ± 0.05	0.11 ± 0.03
Lacrimal glands	0.51 ± 0.23	0.39 ± 0.19	0.05 ± 0.04	0.01 ± 0.01	0.00 ± 0.00
Tumor:Blood	1.11 ± 0.45	3.60 ± 1.24	37.2 ± 4.22	550 ± 108	1714 ± 320
Tumor:Muscle	14.5 ± 4.46	52.2 ± 17.4	275 ± 35.3	886 ± 181	1825 ± 415
Tumor:Kidney	2.39 ± 0.99	4.89 ± 1.58	7.06 ± 0.86	14.6 ± 2.72	24.9 ± 5.16
Tumor:Salivary gland	7.60 ± 2.89	24.5 ± 5.95	69.3 ± 15.8	144 ± 35.6	284 ± 87.9
Blood:Salivary gland	6.93 ± 0.33	7.09 ± 1.45	1.88 ± 0.42	0.26 ± 0.06	0.17 ± 0.04

Table S8: Radiation dose (mGy/MBq) of [^{177}Lu]Lu-HTK03170, [^{177}Lu]Lu-HTK03149 and [^{177}Lu]Lu-PSMA-617 calculated from unit density sphere models for the LNCaP tumors.

Sphere/Tumor volume (mL)	[^{177}Lu]Lu-HTK03170	[^{177}Lu]Lu-HTK03149*	[^{177}Lu]Lu-PSMA-617*
0.01	5.02E+05	7.53E+04	3.07E+04
0.1	5.25E+04	7.87E+03	3.21E+03
0.5	1.07E+04	1.60E+03	6.51E+02
1	5.38E+03	8.06E+02	3.28E+02
2	2.70E+03	4.05E+02	1.65E+02
4	1.36E+03	2.03E+02	8.28E+01
6	9.06E+02	1.36E+02	5.53E+01
8	6.80E+02	1.02E+02	4.15E+01
10	5.45E+02	8.17E+01	3.33E+01
20	2.74E+02	4.10E+01	1.67E+01
40	1.37E+02	2.06E+01	8.38E+00
60	9.17E+01	1.37E+01	5.60E+00
80	6.90E+01	1.03E+01	4.21E+00
100	5.52E+01	8.28E+00	3.37E+00
300	1.86E+01	2.79E+00	1.13E+00
400	1.40E+01	2.10E+00	8.54E-01
500	1.12E+01	1.68E+00	6.85E-01
600	9.37E+00	1.40E+00	5.72E-01
1000	5.66E+00	8.48E-01	3.45E-01
2000	2.87E+00	4.29E-01	1.75E-01
3000	1.92E+00	2.88E-01	1.17E-01
4000	1.45E+00	2.17E-01	8.86E-02
5000	1.17E+00	1.75E-01	7.12E-02
6000	9.76E-01	1.46E-01	5.96E-02

*The calculated dosimetry data of [^{177}Lu]Lu-HTK03149 and [^{177}Lu]Lu-PSMA-617 has been previously reported in Kuo H-T, et al. Theranostics 2022; 12: 6179-6188.

Table S9: Radiation doses (mGy/MBq) of [177Lu]Lu-HTK03170, [177Lu]Lu-HTK03149 and [177Lu]Lu-PSMA-617 calculated for the major organs of 25-g mice using the OLINDA software.

Kinetics value [MBq-h/MBq]				Organ doses [mGy/MBq]			
Source organ	[177Lu]Lu-HTK03170	[177Lu]Lu-HTK03149*	[177Lu]Lu-PSMA-617*	Target organ	[177Lu]Lu-HTK03170	[177Lu]Lu-HTK03149*	[177Lu]Lu-PSMA-617*
Brain	2.55E-02	2.23E-03	6.64E-03	Brain	1.98E+01	2.35E+00	1.79E+00
Large intestine contents	1.12E-01	9.24E-02	8.80E-03	Large intestine	3.27E+01	1.59E+01	2.42E+00
Small intestine	3.35E-01	2.76E-01	2.63E-02	Small intestine	3.21E+01	1.57E+01	2.08E+00
Stomach	1.32E-02	8.07E-03	2.34E-04	Stomach wall	4.00E+01	1.57E+01	1.51E+00
Heart	7.94E-02	1.94E-02	3.28E-03	Heart	4.55E+01	9.38E+00	1.90E+00
Kidneys	1.33E+00	2.41E-01	8.23E-01	Kidneys	3.74E+02	6.72E+01	2.23E+02
Liver	1.24E+00	3.91E-01	6.38E-02	Liver	7.50E+01	2.09E+01	3.84E+00
Lungs	6.60E-02	1.72E-02	4.85E-03	Lungs	6.90E+01	1.62E+01	4.46E+00
Pancreas	4.12E-02	1.79E-02	4.76E-03	Pancreas	3.01E+01	7.72E+00	3.60E+00
Cortical bone	1.91E-01	7.60E-02	8.28E-03	Skeleton	2.93E+01	7.45E+00	1.25E+00
Spleen	1.04E-01	2.30E-02	1.41E-02	Spleen	9.14E+01	1.89E+01	1.15E+01
Testes	9.21E-02	1.29E-02	4.23E-03	Testes	6.22E+01	8.72E+00	3.04E+00
Thyroid	5.15E-03	2.03E-03	4.01E-04	Thyroid	4.31E+01	1.29E+01	2.78E+00
Urinary bladder	1.11E+00	8.14E-01	8.04E-01	Urinary bladder wall	8.08E+02	5.84E+02	5.76E+02
Remainder of the body	4.23E+00	5.31E-01	1.68E-01	Remainder of the body	3.18E+01	8.95E+00	6.89E+00

*The calculated dosimetry data of [177Lu]Lu-HTK03149 and [177Lu]Lu-PSMA-617 has been previously reported in Kuo H-T, et al. Theranostics 2022; 12: 6179-6188.

(A)

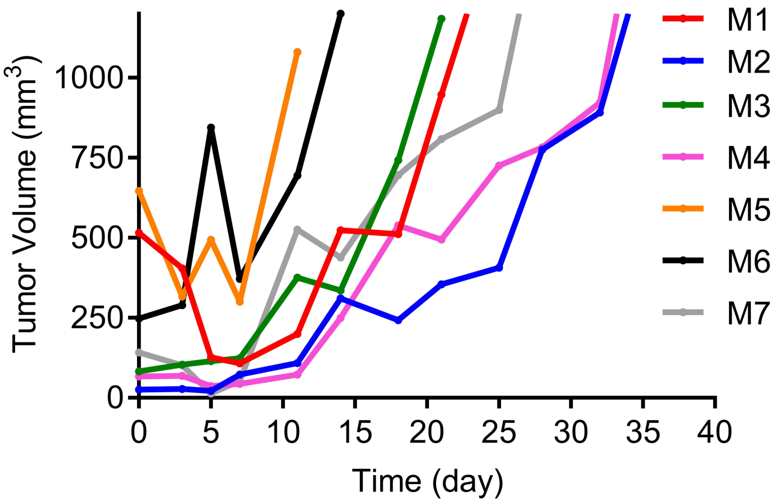

(B)

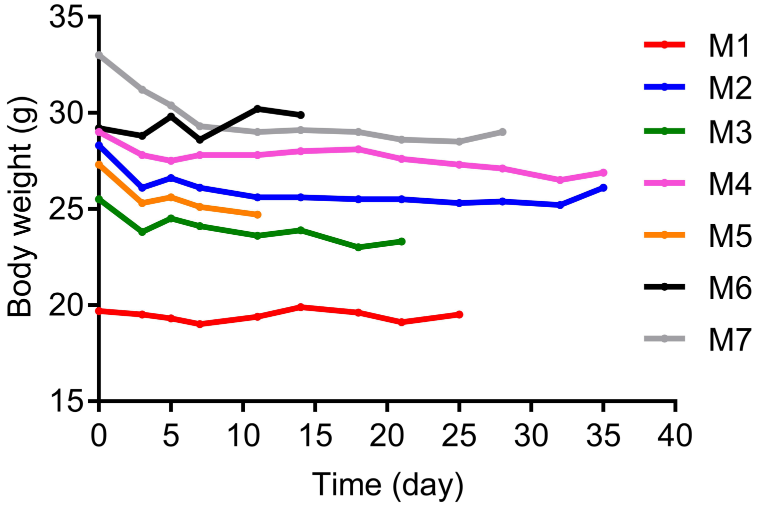

Figure S1: Changes of (A) tumor volume and (B) body weight over time after mice were treated with PBS.

(A)

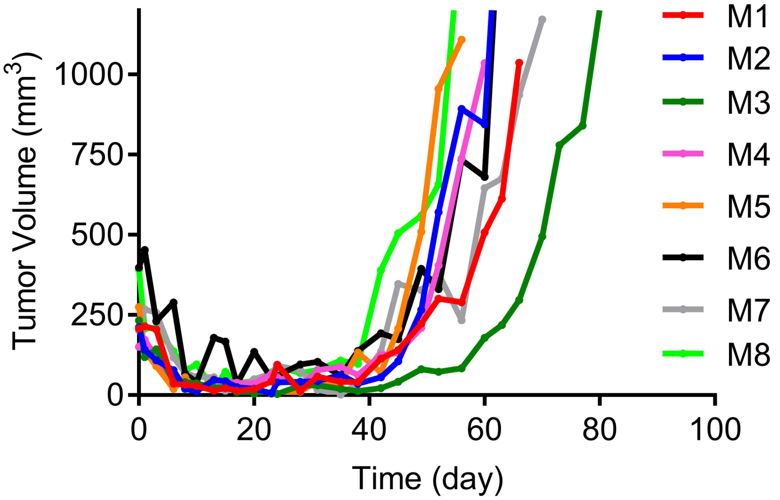

(B)

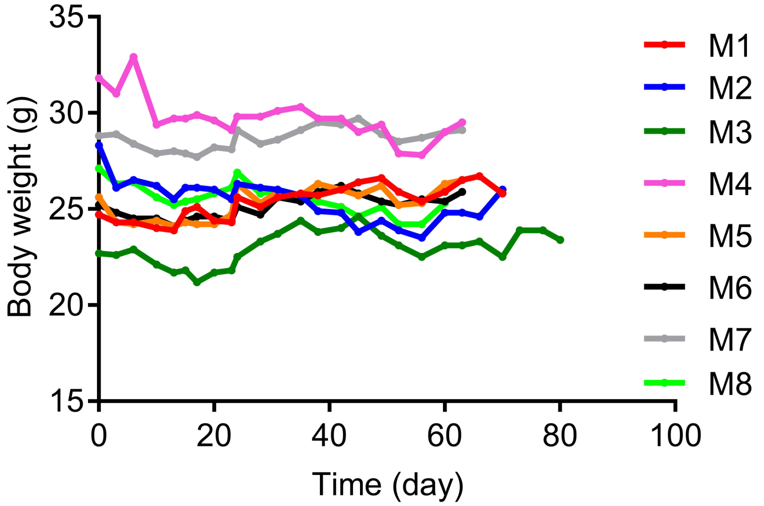

Figure S2: Changes of (A) tumor volume and (B) body weight over time after mice were treated with 37 MBq of [^{177}Lu]Lu-PSMA-617.

(A)

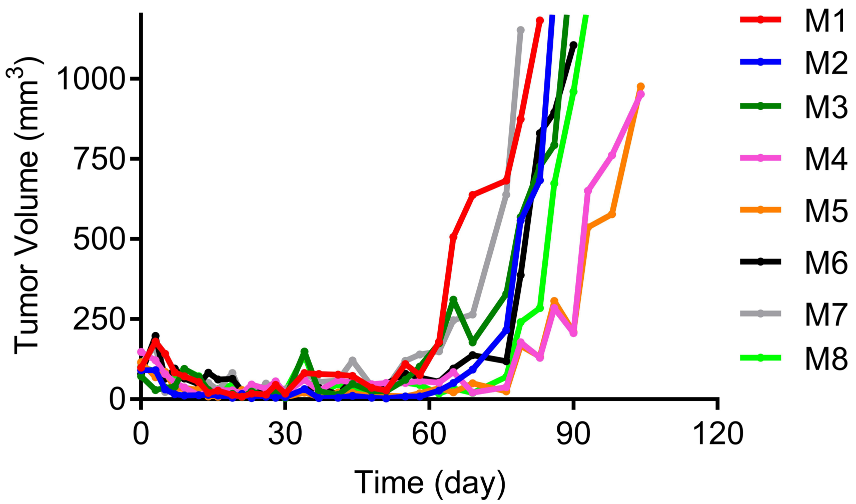

(B)

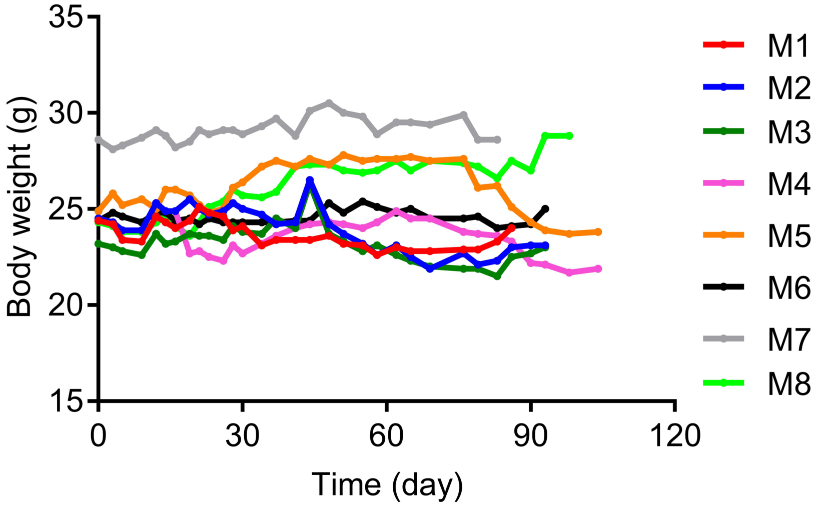

Figure S3: Changes of (A) tumor volume and (B) body weight over time after mice were treated with 9.3 MBq of [^{177}Lu]Lu-HTK03170.

(A)

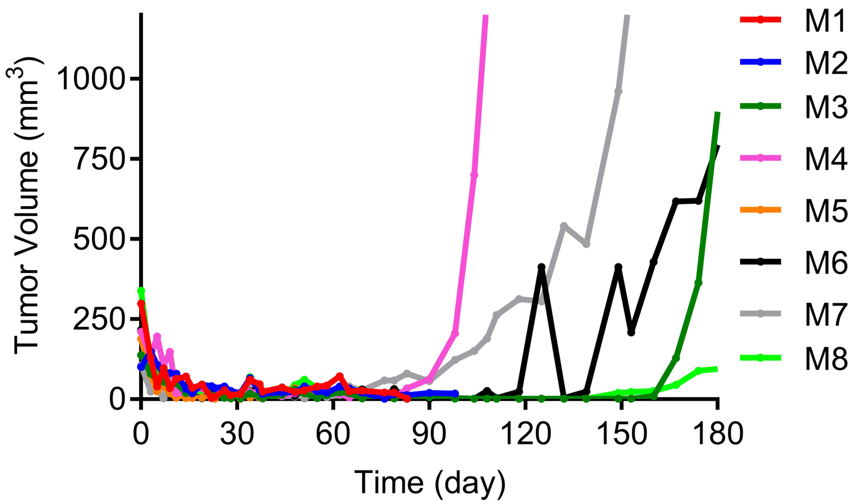

(B)

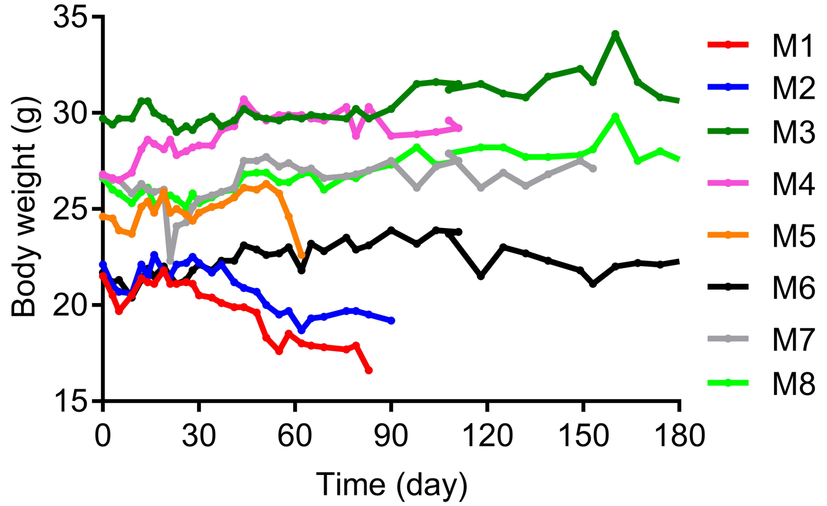

Figure S4: Changes of (A) tumor volume and (B) body weight over time after mice were treated with 18.5 MBq of [^{177}Lu]Lu-HTK03170.

(A)

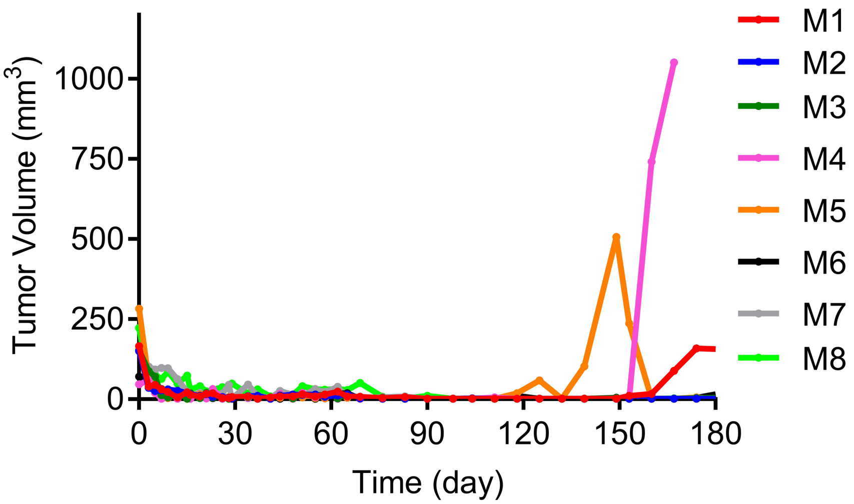

(B)

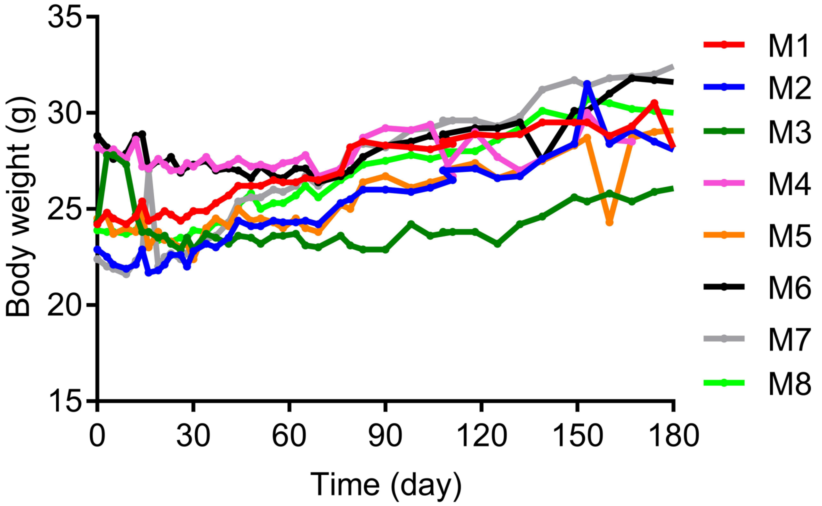

Figure S5: Changes of (A) tumor volume and (B) body weight over time after mice were treated with 37 MBq of [^{177}Lu]Lu-HTK03170.
